# Coupling of PARP1-mediated chromatin structural changes to transcriptional RNA polymerase II elongation and cotranscriptional splicing

**DOI:** 10.1186/s13072-019-0261-1

**Published:** 2019-02-18

**Authors:** Elena A. Matveeva, Qamar M. H. Al-Tinawi, Eric C. Rouchka, Yvonne N. Fondufe-Mittendorf

**Affiliations:** 1 Department of Molecular and Cellular Biochemistry, University of Kentucky, Lexington, KY 40536 USA; 2 Alfaisal University, Al Maather’ Riyadh, 12714 Saudi Arabia; 3 Kentucky Biomedical Research Infrastructure Network Bioinformatics Core, 522 East Gray Street, Louisville, KY 40292 USA; 4 Department of Computer Engineering and Computer Science, University of Louisville, Louisville, KY 40292 USA

**Keywords:** Splicing, RNA polymerase II, Epigenetics, Poly(ADP)ribose polymerase, Polymerase elongation, Nucleosomes, Chromatin

## Abstract

**Background:**

Recently, we showed that PARP1 is involved in cotranscriptional splicing, possibly by bridging chromatin to RNA and recruiting splicing factors. It also can influence alternative splicing decisions through the regulation of RNAPII elongation. In this study, we investigated the effect of PARP1-mediated chromatin changes on RNAPII movement, during transcription and alternative splicing.

**Results:**

We show that RNAPII pauses at PARP1–chromatin structures within the gene body. Knockdown of PARP1 abolishes this RNAPII pausing, suggesting that PARP1 may regulate RNAPII elongation. Additionally, PARP1 alters nucleosome deposition and histone post-translational modifications at specific exon–intron boundaries, thereby affecting RNAPII movement. Lastly, genome-wide analyses confirmed that PARP1 influences changes in RNAPII elongation by either reducing or increasing the rate of RNAPII elongation depending on the chromatin context.

**Conclusions:**

These studies suggest a context-specific effect of PARP1–chromatin binding on RNA polymerase movement and provide a platform to delineate PARP1’s role in RNA biogenesis and processing.

**Electronic supplementary material:**

The online version of this article (10.1186/s13072-019-0261-1) contains supplementary material, which is available to authorized users.

## Introduction

PARP1 also known as ARDT1 belongs to family of proteins known as ADP-ribosylases. This group of enzymes, up to 17 in humans, have varying degrees of homology but a highly conserved PARP catalytic domain. These proteins use NAD+ as a substrate to catalyze the addition of ADP-ribose moiety(ies) onto target proteins, hence the name ADP-ribosyltransferase. Within this family, only PARP1 and PARP2 can build “poly”-ADP-ribose polymers, while the others are capable of adding only a monomeric ADP-ribose to proteins.

PARP1 is the most studied of this family of proteins for which multiple functions been described, which implies pleiotropic functional characteristics. PARP1 is well known for its role in DNA-repair, genome integrity, and cell death [1]. It also adds poly-ADP-ribose (PAR) onto several proteins involved in NAD+ metabolism [2]. Additionally, for innate immunity, DNA damage, or metabolic stress, PARP1 can act as a coactivator of NF-κB transcription factors, contributing to the transcription of a subset of NF-κB target genes [3]. Increasingly, the role of PARP1 in modulating chromatin structure to regulate gene expression is being recognized. PARP1 adds PAR residues (PARylates) onto histones [4], which opens the chromatin structure and enables gene activation. In support of this function of maintaining active transcription, several genome-wide studies show PARP1 to be associated with active gene promoters [5, 6], and competing with the repressive histone H1 [7, 8] to elicit differential gene expression outcomes. However, while it is clear that PARP1 is important in gene activation, other studies have shown that depletion of PARP1 also results in gene repression [7–11], suggesting that PARP1 most likely acts in a context-specific manner.

Regulation of gene expression occurs at both the transcriptional initiation and splicing levels, with chromatin structure influencing both processes. Interestingly, while the role of chromatin in transcription has been studied significantly, the role of chromatin in splicing is just emerging. The recent discovery that splicing, or the decision of a particular region to be spliced, occurs cotranscriptionally while the nascent mRNA is still tethered to chromatin, developed into the cotranscriptional splicing hypothesis [12–14]. Indeed, changes to the epigenome that mediate chromatin structural integrity have been implicated in alternative splicing regulation. For instance, DNA methylation and histone modifications demarcate exon–intron boundaries [13, 15–19] that regulate splicing decisions. It is, therefore, possible that in regulating chromatin structure, PARP1 might also play a role in this process. Indeed, our previous study demonstrated a functional role for PARP1 and PARylation in the regulation of pre-mRNA splicing [5]. We showed that PARP1 binds to nucleosomes at target exon/intron boundaries, mediating specific splicing decisions. In addition, we demonstrated that knockdown of PARP1 or inhibition of its PARylation activity resulted in changes in specific alternative splicing patterns. Interestingly, splicing products in PARP1 knockdown (KD) cells versus PARylation-inhibited cells were not similar, possibly suggesting that the effects of PARP1 on chromatin binding are direct while its PARylation activity is indirect. We therefore hypothesized that modulation of chromatin structure by PARP1 directly affects splicing decisions, while its PARylation activity could regulate splicing through activation of splicing factors [20] and/or through PARylation of histones [1, 21, 22] to open up the chromatin structure. However, a clear understanding of how PARP1 regulates alternative splicing is unknown.

Two non-mutually exclusive models have been hypothesized to explain how chromatin structure or factors that modulate chromatin structure, regulate alternative splicing. (1) The kinetic model proposes that chromatin structure regulates the speed of polymerase elongation to influence splicing outcomes [23]. (2) The adaptor/recruitment model proposes that chromatin or its associated factors recruit splicing factors, bridging the chromatin structure and the nascent mRNA [24–26]. PARP1 could act in both models: PARP1–chromatin binding is well established, and in addition to this function, we showed that PARP1–chromatin binds to splicing factor 3B1 (SF3B1), a U2 spliceosomal member [5], and might therefore act as an adapter, bringing the splicing complex (with SF3B1) to RNA. As a further step, we showed that PARP1 binds RNA and identified the PARP1-mRNA binding landscape [11]. In summary, these data support the adapter model for PARP1 to regulate splicing. However, it is not implausible that PARP1 also could regulate the rate of polymerase elongation, especially as PARP1 has been shown to be involved in polymerase pausing at the promoters of heat shock genes [27]. We therefore hypothesized that if PARP1 plays a role in polymerase pausing at the transcription level (promoter region), it may also play a role in chromatin structure that pauses RNAPII elongation along the gene body for splicing regulation. In the current study, we propose that PARP1 also works as an RNAPII regulator through mutual interdependence of splicing and transcription elongation. Using S2 *Drosophila* cells as a convenient model (*Drosophila* contains only one PARP1 gene), we tested the effect of PARP1 on the RNAPII elongation rate and cotranscriptional splicing.

## Results

### PARP1 is involved in mRNA splicing

**Fig. 1:**
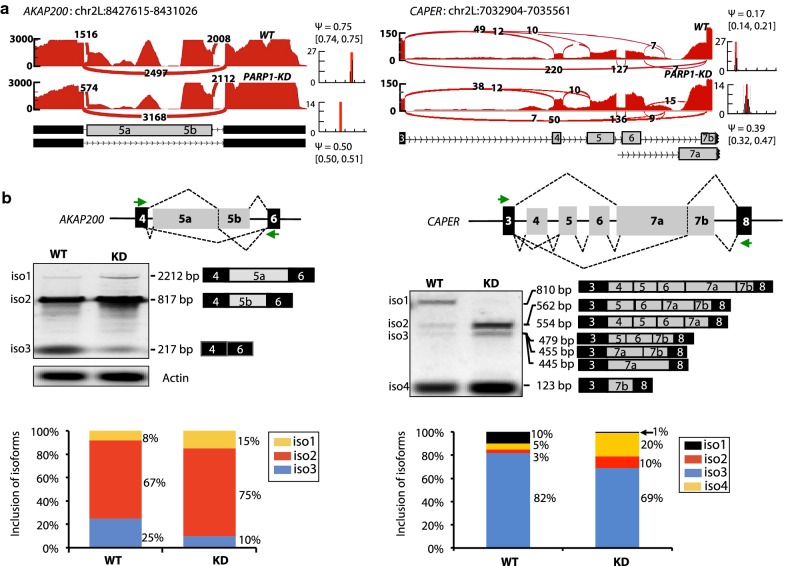
PARP1 depletion alters splicing decisions. **a** Sashimi plots showing changes in the splicing decisions due to PARP1 depletion in RNA-seq genome-wide analyses for *AKAP200* and *CAPER*. **b** PCR products with exon junction spanning primers validate splicing changes in *AKAP200* and *CAPER.* Agarose gel images and percentage of exons inclusion show the difference in splicing product between non-treated (WT) and PARP1 knockdown (KD) cells. Actin is shown as a PARP1 non-target gene. Additionally, the percentage of each isoform included is calculated as a percentage of all transcripts amplified by PCR set to a 100%

We previously showed that PARP1 KD in *Drosophila* S2 cells results in changes in alternative splicing of several genes [5]. Our goal in this study is to understand mechanistically how PARP1 modulates chromatin structure to regulate splicing decisions. We chose to analyze this mechanism in depth at two genes—*AKAP200* (hence called *AKAP*) and *CAPER*—because we found that (1) PARP1 binds to nucleosomes within their gene bodies and (2) PARP1 depletion correlated with changes in the splicing decisions observed through RNA-seq genome-wide analyses [5] (Fig. 1a). We used RNA interference to deplete PARP1 in S2 Drosophila cell lines (Additional file 1: Fig. S1), to assess whether PARP1 binding on *AKAP* and *CAPER* exon–introns reflects a direct role for PARP1 in alternative splicing decisions at these genes. We then performed PCR with exon junction spanning primers to validate the splicing changes (Fig. 1b). These results validated the genome-wide studies [5] and showed that in the absence of PARP1, differentially spliced transcripts for *AKAP* and *CAPER* were produced. Furthermore, a second siRNA (siRNA2) targeting a different region of PARP1 (Additional file 1: Fig. S1A and B) confirmed these results (Additional file 1: Fig. S2). To test whether this effect is due to PARP1 directly or its enzymatic activity, we inhibited its PARylation using PJ34 (Additional file 1: Fig. S1C) and showed that PARylation inhibition effected no changes in splicing at these two target genes (Additional file 1: Fig. S2).

### PARP1–chromatin structure influences RNA polymerase elongation

We next tested whether PARP1 regulates splicing through regulation of the rate of RNAPII elongation. For this, we used our genome-wide data of PARP1 nucleosome occupancy (GSE56120) in *Drosophila* S2 cells with PRO-seq data [28] (GSE42117) of transcriptionally engaged RNA polymerase. Analyses of these binding profiles showed that PARP1 and engaged RNAPII are in close proximity within gene bodies. Indeed, we observe a shift ~ 25 bp downstream of the PARP1 signal relative to the RNAPII signal (shown as metagene plots in Additional file 1: Fig. S3). These data suggest that PARP1 may be involved in RNAPII elongation stalling. Next, we investigated if these binding profiles are true in our genes of interest above.

**Fig. 2:**
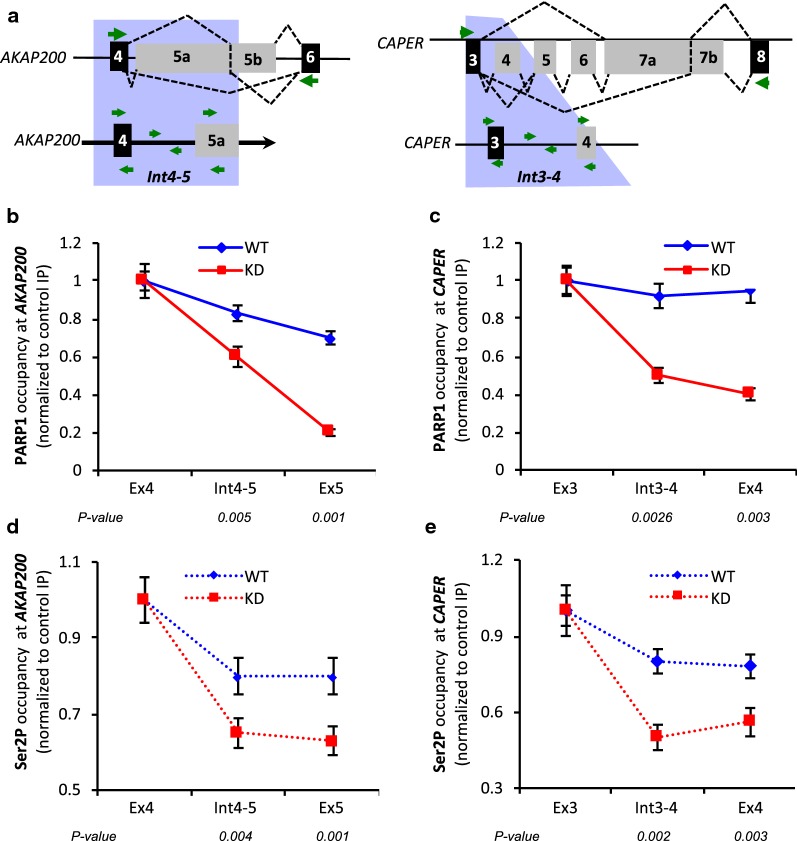
PARP1 and RNAPII ChIP-qPCR for PARP1 target genes *AKAP200* and *CAPER.* **a** Diagram of constitutive (black boxes) and alternative (gray boxes) exons of a wider region (upper) and zoomed in region (lower) for *AKAP200* and *CAPER* genes. Green arrows depict primer locations. **b**, **c** Showing the relative occupancy of PARP1 in wild type (WT, blue line) and PARP1 knockdown (KD, red line) cells for *AKAP200* and *CAPER* at the alternative exon relative to presiding constitutive exon. **d**, **e** Showing RNAPII occupancy (elongation form, Ser2P) for *AKAP200* and *CAPER.* All experiments were performed in triplicate, and results are presented as mean ± SD (*p value < 0.05). Statistical significance was tested by Student’s *t* test method

The processivity of RNAPII depends on the phosphorylation state of its carboxy terminal domain (CTD). In particular, the transition between initiation-pausing and productive elongation is marked by phosphorylation on Ser5 and Ser2, respectively [29–31]. We therefore asked whether PARP1 influences the recruitment of different forms of phosphorylated RNAPII to exonic regions of our target genes, *AKAP* and *CAPER*. Three antibodies that bind specific phosphorylation states of RNAPII were used: (1) 4H8, which recognizes Ser5 phosphorylation (marks initiating and first regions of the gene)—hence referred to as Ser5; (2) H5, which recognizes Ser2 phosphorylation is found mainly in the gene body and toward the 3′ end of the gene. This form is known as the transcriptionally engaged and elongating form of RNAPII—hence referred to as Ser2P; (3) 8WG16, which recognizes hypo-phosphorylated RNAPII found at pre-initiation sites. The 8WG16 antibody has been reported to sometimes also recognize Ser5-phosphorylated RNAPII and could be used to determine total RNAPII. We therefore performed ChIP-qPCR using these antibodies on cross-linked chromatin from wild type (WT) and PARP1 knockdown (KD) cells and analyzed the occupancy of the various forms of RNAPII on the two PARP1 target genes—*AKAP* and *CAPER* (Fig. 2 and Additional file 1: Fig: S4). To better assess the correlation between PARP1 reduction and RNAPII occupancy, we calculated the ratio between the occupancies of these polymerase forms and PARP1 at three locations: (1) the immediate preceding constitutive exon; (2) the intervening intron; (3) the proceeding alternative exon of these genes as shown in Fig. 2a. We call this the ‘travelled’ index as it determines the ratio of PARP1 (or RNAPII) occupancy at the proceeding intron or alternative exon, relative to the preceding constitutive exon.

To begin, the occupancy of PARP1 was measured. In WT cells, at the *AKAP* gene, a 22% reduction in the relative occupancy of PARP1 was observed at the alternative exon 5 compared to constitutive exon 4 (Fig. 2b and Additional file 1: Fig. S4A—blue bar). When using the ‘travelled’ index, this represented a steady decline of PARP1 occupancy from the 5′ constitutive exon 4 to proceeding intron 4–5 and was lowest at the 3′ alternative exon 5 (Fig. 2b). In PARP1 depleted conditions (PARP1 KD cells), a reduction of ~ 80% of PARP1 occupancy was measured at alternative exon 5 relative to constitutive exon 4 (Additional file 1: Fig. S4A—red bar). With the travelled index incorporated, this change represented a further decrease of ~ 45% and ~ 80% in PARP1 occupancy at the proceeding intron 4–5 and at alternative exon 5 relative to the constitutive exon (Fig. 4b—red line). A similar trend was observed at the* CAPER* gene though the decrease in the relative amount of PARP1 occupancy from constitutive exon 3 to alternative exon 4 was less pronounced. As expected, there was an additional decrease in PARP1 occupancy at all the tested sites in KD cells (Fig. 2c and Additional file 1: Fig. S4A).

Next, we asked whether the observed changes in PARP1 occupancy correlate with changes in the occupancy of RNAPII forms. Using the Ser2P antibody, which recognizes the transcriptionally engaged, elongating form of RNAPII, we showed that depletion of PARP1 correlated with depletion of this transcriptionally engaged RNAPII at the studied exons. These data corroborated our directly measured results comparing alternative versus constitutive exons and also those measured by the travelled index. In WT cells, as measured through direct comparison of occupancy at alternative exon versus constitutive exon, the *AKAP* gene showed a decrease of ~ 20% (Additional file 1: Fig. S4B). Further supporting these data, the travelled index (Fig. 2d) showed a steady decline of Ser2P from the 5′ constitutive exon toward the 3′ alternative exon. These results were very similar for the *CAPER* gene (Fig. 2e and Additional file 1: Fig. S4B). Interestingly, in PARP1 knockdown cells, Ser2P decreased even further (~ 40% at intron 4–5 and alternative exon 5 compared to constitutive exon 4 at *AKAP*) (Fig. 2d and Additional file 1: Fig. S4B), while this decrease in Ser2P was ~ 50% at *CAPER* gene regions (Fig. 2e and Additional file 1: Fig. S4B). These findings correlated with the observed decrease in PARP1 occupancy at these sites (Fig. 2b, c; Additional file 1: Fig. S4A). Next, we asked if this correlation of PARP1 occupancy is specific for only the elongating Ser2P occupancy. For this, we tested the occupancy of the other phosphorylated forms of RNAPII—the pre-initiating form of RNAPII, also known as hypo-phosphorylated RNAPII (8WG16), and Ser 5 (4H8). In WT cells, the occupancy of 8WG16 at alternative exons over constitutive exons was reduced by 50% for both *AKAP* and *CAPER* genes, respectively (Additional file 1: Fig. S4C—blue bars). On the other hand, in KD cells, we observed an increase in the presence of this polymerase form (Additional file 1: Fig. S4C—red bars). Profiling of 4H8, which measures Ser5 which is found at TSSs and gene bodies, showed a slight increase at the alternative exon 5 of *AKAP* compared to the constitutive exon 4. For *CAPER*, we observed a large and significant decrease of ~ 80% occupancy of Ser5 (Additional file 1: Fig. S4D—blue bars). In KD cells, *AKAP* exhibited an increase in this polymerase form while *CAPER* showed no significant difference in occupancy compared to WT cells (Additional file 1: Fig. S4D—red bars). Finally, analysis of the occupancies of PARP1 and the various RNAPII were recapitulated in cells treated with a second siRNA (siRNA2), thus confirming the effect of PARP1 on the occupancy of RNAPII. In contrast, inhibition of PARylation showed no differences in PARP1 or in the occupancies of the different RNAPII forms when compared to WT cells (Additional file 1: Fig. S5). These data therefore show that PARP1 occupancy and not its PARylation activity exerts an effect on the occupancy of elongating polymerase.

### PARP1 depletion disrupts chromatin state and structure

**Fig. 3:**
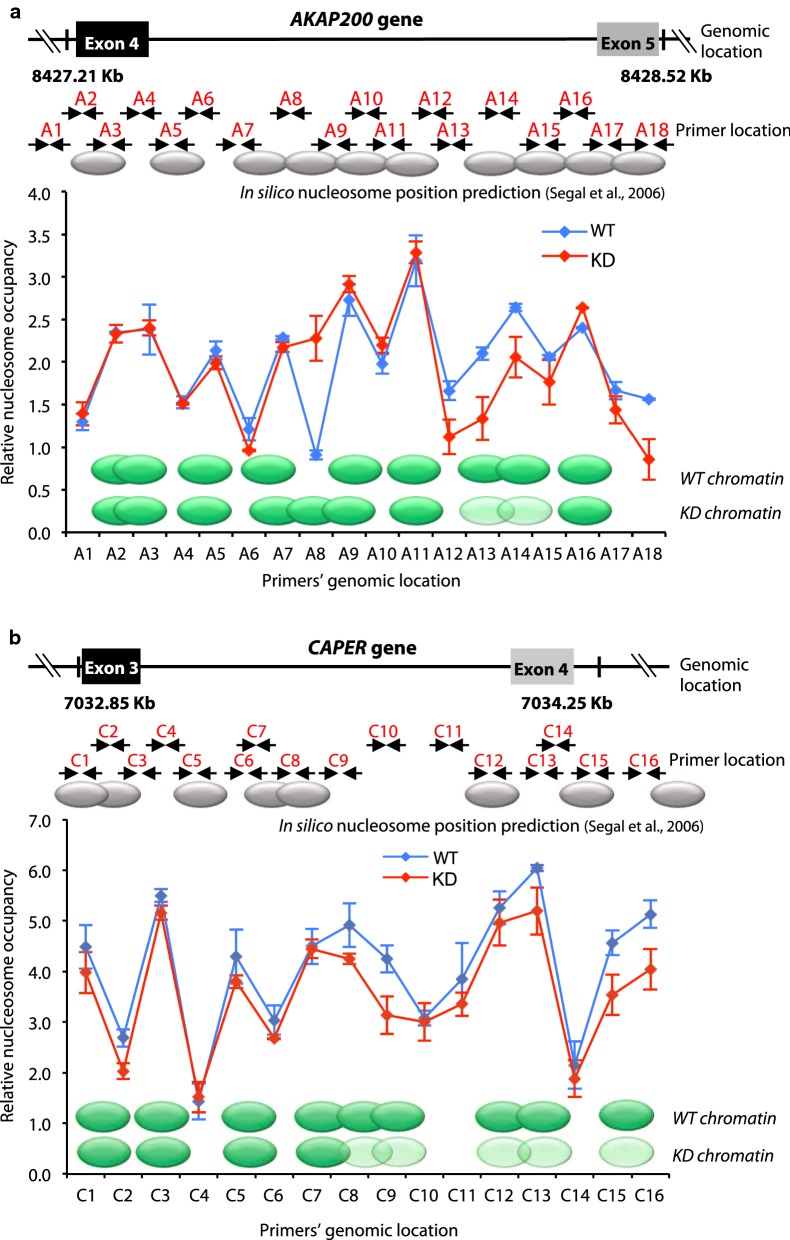
Analysis of nucleosome architecture. The positions of nucleosomes in non-treated cells (WT, blue line) versus PARP1 knockdown (KD, red line) for the *AKAP200* (**a**) and *CAPER* (**b**) genes are distinctive. For each gene, the top panel shows gene region and the predicted nucleosome location (gray ovals) based on sequence alone [34]. The bottom panel demonstrates the nucleosome position and occupancy (green ovals) obtained by nucleosome scanning analysis (see Methods). Depletion of PARP1 shifts nucleosome positioning and occupancy. Light green ovals depict reduction in nucleosome occupancy, while fuzzy nucleosomes are represented as nucleosomes overlying each other. All experiments were done in triplicate, and results are presented as mean ± SD (results were considered significant as determined by Student’s *t* test method: *p* value < 0.05)

Chromatin context can affect the rate of RNAPII elongation, which in turn, would regulate alternative splicing. After confirming the relationship between PARP1 and RNAPII pausing, we next investigated the type of chromatin context mediated by PARP1 at these alternative exon sites. For this, we mapped the nucleosomes across *AKAP* and *CAPER* genes using the nucleosome walking method [32]—a low-resolution technique, which allows gene-specific high-resolution mapping of nucleosome positions along a stretch of DNA [33]. Chromatin is digested with micrococcal nuclease (MNase) to yield mostly mononucleosomal fragments and is then subjected to quantitative real-time PCR (qRT-PCR—see Methods). First, we predicted nucleosome locations based on sequences alone [34] (Fig. 3a, b—top panels). Then, primers were designed to amplify about 80–100-bp-sized amplicons that overlapped by 20–40 bp, tiling across the selected loci of the *AKAP* and *CAPER* genes. In this technique, amplification of a product indicates the presence of a protected mononucleosome, while the lack of amplification signifies open chromatin susceptible to MNase digestion. Nucleosome positions and strength of nucleosome occupancy were then calculated using the fold change between MNase-treated samples and undigested genomic DNA at an equivalent DNA input concentration (see Methods).

Based on this method, stable and highly reproducible profiles of nucleosomes were observed across the target gene region (Fig. 3), with clear differences in the nucleosome profile between WT and PARP1 KD cells. For the *AKAP* gene, two clear observations were made: (1) There is a strong nucleosome in PARP1 KD cells, mapped by primer A8, which was previously absent in WT cells. (2) At the genomic locations mapped by primers A13 to A15, a reduction in nucleosome occupancy in PARP1 KD cells was seen, with a shift of the nucleosomes toward the A16 position (Fig. 3a). For the *CAPER* gene, we observed a reduction in nucleosome occupancy just before the alternate exon 4 (mapped by primers C8–C10) and in the region containing the alternative exon 4 (mapped by primers C12–C16) (Fig. 3b). Here too, PARP1 knockdown using siRNA2 produced similar results for nucleosome repositioning as seen in cells transfected with PARP1 siRNA1, while PARylation-inhibited cells exhibited no changes in nucleosome positioning when compared to WT cells (Additional file 1: Fig. S6). Since these PARP1-mediated nucleosome rearrangements occur right before the alternate exon, we posit that PARP1 maintains a chromatin structure that would be amenable to transcription elongation by RNAPII in the absence of PARP1.

### PARP1 occupancy displays interplay of selective acquisition of histone methylation at genic regions

**Fig. 4:**
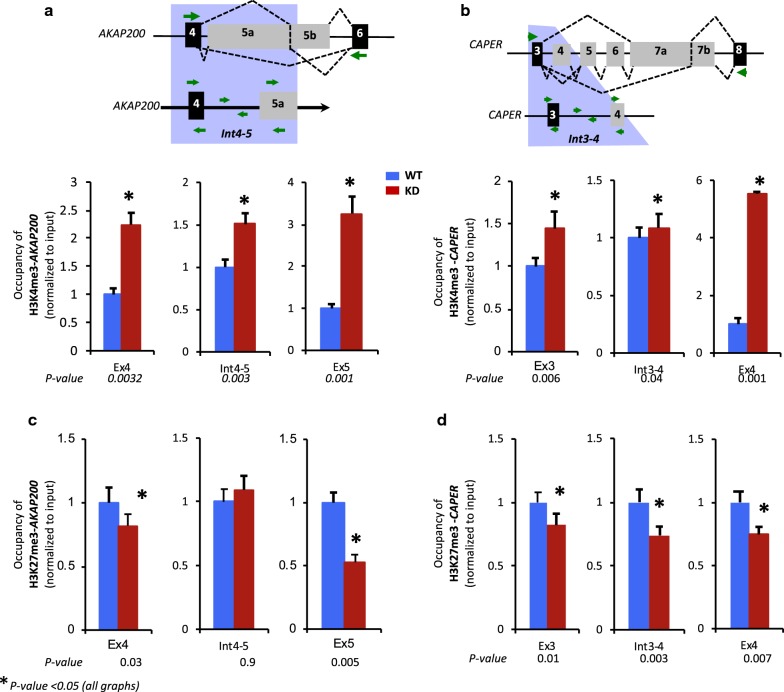
Knockdown of PARP1 induces dynamic changes in the occupancy of histone marks. The occupancy of activating histone marks (H3K4me3; lower panels of **a**, **b**) at *AKAP200* (**a**) and *CAPER* (**b**), and the occupancy of the repressive histone marks (H3K27me3) at *AKAP200* (**c**) and *CAPER* (**d**) were evaluated by ChIP-qPCR for PARP1 target genes. All experiments were performed in triplicate, and results are presented as mean ± SD (*p* value < 0.05). Statistical significance was tested by Student’s *t* test method

Several histone marks have been implicated in alternative splicing [20]. Given that our previous data showed interplay between PARP1 and certain histone marks [5], we then sought to determine whether interplay of PARP1 with specific histones could drive the observed chromatin rearrangement and transcriptional elongation machinery at the studied regions. Typically, paused gene regions are marked by bivalent histone marks. In view of this, we used ChIP-qRT-PCR, to measure the occupancy for both the activating mark, H3K4me3, and repressive mark, H3K27me3, in regions that showed the most change in nucleosome structure—regions mapped by primers A14 for *AKAP* and C8 for *CAPER* and its surrounding exons (Fig. 3). In WT cells, at both genes, H3K4me3 and H3K27me3 were found at all sites tested (preceding constitutive exon, intervening intron, and alternative exon) with varying levels (Fig. 4). The presence of both the activating H3K4me3 and repressive mark H3K27me3 is indicative of a poised gene region. To further assess whether there is interplay between these histone marks and PARP1, we investigated whether their occupancy is changed in the absence of PARP1. Knockdown of PARP1 resolved this bivalency of H3K4me3 and H3K27me3 marks to H3K4me3. At both of these genes, there was an increase in H3K4me3 occupancy (Fig. 4a for AKAP and 4c for CAPER) and a decrease in H3K27me3 occupancy (Fig. 4b for AKAP and 4D for CAPER). The resultant net gain of H3K4me3—an active histone mark—possibly opens up the chromatin structure allowing for the passage of transcription machinery. These data were recapitulated in cells treated with siRNA2 (Additional file 1: Fig. S7—red bars vs. blue bars), while PARylation-inhibited cells showed no difference relative to WT cells (Additional file 1: Fig. S7—green bars vs. blue bars). In summary, our data are consistent with a model in which binding of the PARP1 mediates or is mediated by specific histone modifications. Additionally, the effect of PARP1 on chromatin structure (nucleosome positioning and occupancy of histone PTMs) is instigated by the direct presence of PARP1 and not its PARylation activity. Thus, at the sites of alternative splicing, PARP1 could play a dual role in stimulating the release of RNAPII pausing and recruiting chromatin modifications that facilitate its release from the paused state.

### PARP1 influences RNAPII elongation genome-wide

**Fig. 5:**
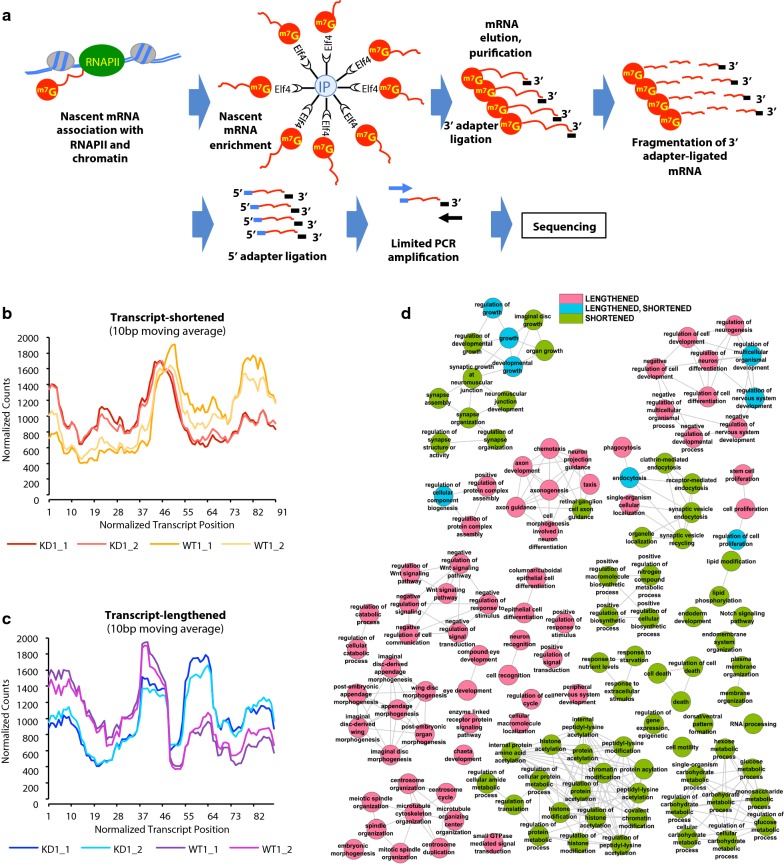
NET-seq results. **a** A schematic representation of the NET-Seq Illumina library preparation. **b** Metagene analysis of NET-Seq at different genomic regions for wild type (WT) and PARP knockdown (KD) cells reveals shortened transcripts (**b**: orange lines—WT, red lines—KD) and lengthened transcripts (**c**): purple and violet lines—WT, blue lines–KD). **d** Visualization of gene ontology: biological process categories of PARP1 effecting of RNAPII activity

The observations of the direct effect of PARP1 binding on chromatin structure and histone modification occupancy prompted us to ask whether PARP1 could influence the release of RNAPII from pause sites. We used a modified 3′NT-seq method [35] and the NET-seq method [36] to map the positions of elongating and arrested RNAPII complexes at nucleotide resolution (Fig. 5a) in the presence and absence of PARP1. The 3′NT method effectively isolates mRNA from RNA-RNAPII–chromatin complexes. The presence of the m7G cap on RNAPII transcripts within the first 20–30 nucleotides of transcription allows for the immunoprecipitation of nascent mRNAs using the GFP-elF4E protein (which binds to the m7G cap) bound on magnetic beads. These captured mRNAs were then eluted from beads, purified, and ligated to the 3′ adapter used in Illumina sequencing. Next, the RNAs were fragmented to not only decap the captured 5′ ends of the mRNAs, but also to reduce the size of the mRNAs to ~ 35–100 nucleotides. Fragmented mRNAs were then size selected on a denaturing gel. Next, the 5′ phosphate groups were removed enzymatically, allowing for the ligation of the 5′ sequencing adapter. cDNA libraries were then prepared, and limited PCR amplification with primers that bind to both the 5′ and 3′ adapters was performed. This allowed for the capture and sequencing of only mRNA fragments with both the 5′ end (end after fragmentation) and the original 3′ end of the nascent mRNAs. After PCR, samples were size selected on a 3.3% NuSieve agarose gel. These fragments were treated and analyzed separately, gel purified, and subjected to Illumina high-throughput 50 bp paired-end sequencing. Two biological replicates for WT and PARP1-KD cells were sequenced, generating ~ 30–100 nt reads for each 3′NT-seq sample (LW1 and its corresponding HW1; LW2 and its corresponding HW2). A total of 780 million reads were sequenced, 101 million of which mapped uniquely (i.e., after removing multi-mapped reads and potential PCR duplicates) to the *Drosophila* genome (Dm6) after additional filtering steps.

In 3′NT-seq experiments, the sequenced read density reflects the abundance of the transcript and the 3′ ends of the nascent mRNAs map the RNAPII position at nucleotide resolution. Thus, assuming there is no degradation, these sequenced RNAs would have the captured 3′ ends of the elongating polymerase just before transcription elongation inhibition by alpha-amanitin. In fact, the resolution afforded by 3′NT-seq and the coverage obtained should provide an in-depth view of genome-wide transcriptional activity. Thus, to begin our analyses, we first compared the reproducibility of the biological replicates using the multiBamSummary tool from DeepTools 2.0 [37]. The biological replicate libraries show strong agreement (Pearson’s coefficient > 0.988), which documents the robustness of our approach. We then compared the sequencing reads normalized by reads per kilobase of transcript, per million mapped reads (RPKM) between WT and PARP1 KD cells. To determine if transcription of specific segments of mRNA genes is targeted by PARP1, we calculated the number of normalized reads for all mRNA genes and divided them into five separate regions: 1000 bases immediately upstream of the start codon (upstream); transcript (from transcription start sites (TSS) to transcription end sites); first 100 bp of the transcript; last 100 bases of the transcript; and 1000 bases downstream of the transcript end (downstream).

A total of 100 bins were created for each region. In the initial global test, using a cutoff of *p* < 0.01, subtle differences between WT and PARP1 KD samples were observed in the bins for the upstream and transcript regions (Additional file 1: Fig. S8A, B). On the other hand, some differences were observed at downstream regions as well as at the early (first 100 nt) and late gene bodies (last 100 nt) due to PARP1 knockdown (Additional file 1: Fig. S8C–E). Next, using the difference in the percentage of reads occurring in the first 50 bins, we filtered the differentially expressed regions into three groups: Group 1: genes with reads that were shortened; Group 2: genes with reads that were lengthened; Group 3: genes with reads that had differential patterns not specifically related with shortening or lengthening. This analysis resulted in a total of 1786 genes, of which 348 (Group 1) and 307 (Group 2) had lengthened and shortened transcripts, respectively, in KD versus WT cells, and the rest were placed in Group 3. We show the top 20 shortened (Additional file 1: Table S1) and lengthened genes (Additional file 1: Table S2). Examples of shortened and lengthened genes are shown in Additional file 1: Fig. S9A and B, respectively.

To better understand the pattern of these lengthened and shortened genes, we also performed metagene analysis of the 3′NT-seq at different genomic regions. We observed a shifting of RNAPII locations, evidenced by average RNAPII densities in the PARP1 KD, which were substantial, often decreasing (shortened) or increasing (lengthened) (Fig. 5b & c), when compared to the same sites in WT cells. In the KD condition, when transcripts have shortened RNAPII profiles, there is a slight shift in the peak of RNAPII location toward the 5′ end of the gene relative to WT (Fig. 5b). For lengthened genes, one main peak (location) of RNAPII was observed in WT cells. In PARP1 depleted cells, there was a decrease in this peak with a concurrent appearance/increase of a new peak located 3′ of this peak (Fig. 5c), resulting in two prominent peaks.

At the upstream regions, in genes with a shortened RNAPII profile, one peak was detected with little to no change in PARP1 KD cells (Additional file 1: Fig. S10A). As for the lengthened genes, several peaks were present. In cells with depleted PARP1, there was a reduction in the peaks at the 5′ region with a concurrent increase in the peaks at the 3′ end (Additional file 1: Fig. S10B). A similar situation occurred for the RNAPII downstream regions with shortened genes (Additional file 1: Fig. S10C). On the other hand, slight differences were detected with lengthened genes, including a stronger peak emerging toward the 5′ of the WT RNAPII positions (Additional file 1: Fig. S10D). At the other gene regions, the first and last 100 bp gene regions, a shortened RNAPII profile can be seen, similar to the downstream regions (Additional file 1: Fig. S10E and G, respectively). With the lengthened genes, there seems to be a flattening and merging of the two RNAPII peaks in PARP1 KD cells for the RNAPII locations within the first and last 100 bp genic regions (Additional file 1: Fig. S10F and H). According to other studies, a fast polymerase is typically associated with an overall flattening of the RNAPII profile in the termination zone replacing the clear drop-off in RNAPII density that occurs in WT cells. Interestingly, such a flattened profile has been associated with reduced pausing [38]. Finally, we asked whether these genes with shortened or lengthened transcript regions are involved in any functional pathways. For this we performed gene ontology: biological process analysis (GO:BP) using categoryCompare [39]. GO:BP analysis results showed that genes with a shortened RNAPII profile were significantly enriched in genes associated with cell processing (Fig. 5d and Additional file 1: Table S3). On the other hand, the genes with a lengthened RNAPII profile were significantly involved with organismal organization (Fig. 5d and Additional file 1: Table S4).

**Fig. 6:**
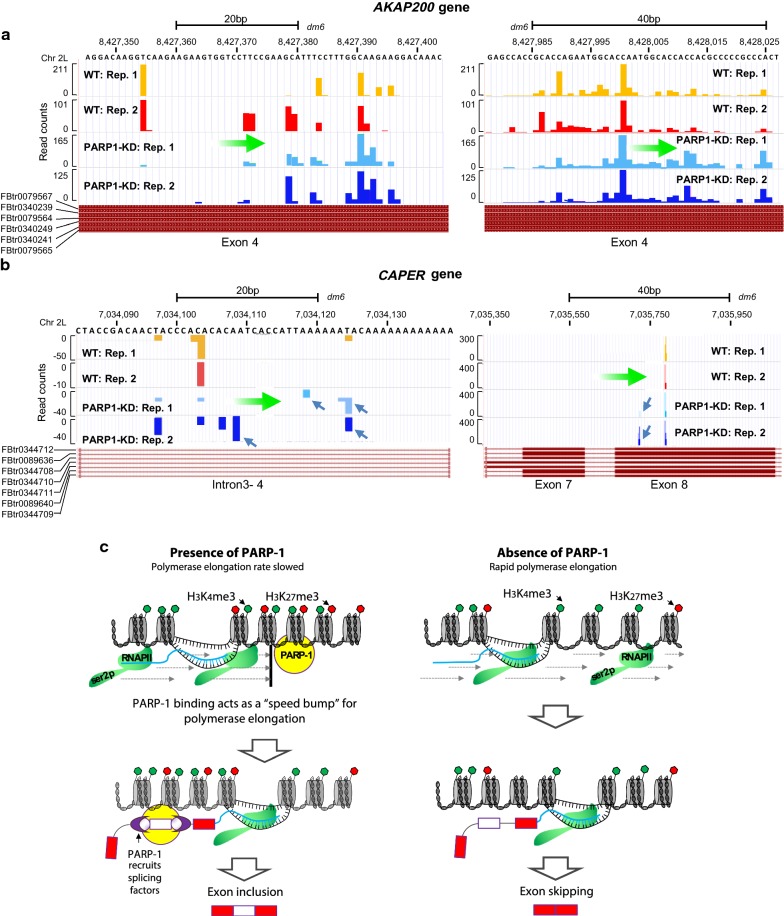
Graphic representations of the RNAPII NET-seq results at (**a**) AKAP and (**b**) CAPER. RNAPII localization signals normalized as NET-seq read count signals. Blue arrows indicate the position the final nucleotide added by RNAPII, while green arrow indicates the movement of the polymerase (**c**). ‘Kinetic model’ of PARP1–chromatin binding effects on RNAPII elongation, with consequences in alternative splicing regulation. PARP1 creates and/or maintains a chromatin structure, which is poised for transcription elongation. This structure enables RNAPII to pause, allowing enough resident time for RNAPII and its associated splicing factors to recognize specific splice sites, resulting in exon inclusion. In the absence of PARP1, the poised state is resolved to a more active chromatin structure, thus no pausing of the fast RNAPII, resulting in exon skipping. Although this model can explain our results of lengthening of the transcripts after PARP1 knockdown, it does not explain shortening of transcripts after knockdown

We also provide NET-seq analyses on our candidate genes, *AKAP* and *CAPER*. Prominent peaks of RNAPII density are seen at specific nucleotides or within narrow regions, possibly indicative of pause sites (locations where RNAPII is detected with high probability). For *AKAP* (Fig. 6a), NET-seq showed a prominent peak at the 5′ of the region, with more RNAPII footprints downstream. Of interest, in KD cells, the main 5′ peak is absent, with more RNAPII footprints downstream compared to WT. Indeed, the last nucleotide added is further along the gene. These results suggest that after the PARP1-mediated block is relieved (through knockdown of PARP1), more RNAPII was able to move downstream to selectively include specific exons over others (Fig. 6a). The situation at the *CAPER gene* is slightly different. NET-seq in KD cells shows an increase in RNAPII footprint within the preceding intronic region (left panel—Fig. 6b), and a slowing down of RNAPII around the proceeding exonic region located at 7,035,750 bp (Fig. 6b). These results of a fast RNAPII elongation at upstream intronic regions and slow down of RNAPII elongation at the proceeding exons, could explain the selective exon skipping seen in PARP1 KD cells (Fig. 1b). Additionally, these results resonate with previous studies showing that slowing down of RNAPII not only results in exon inclusion, but has been implicated in exon skipping as well [40]. In summary, our analyses for specific genes show a clear RNAPII pausing defect (positive and negative) due to PARP1 depletion. Interestingly, these changes in RNAPII elongation occur at the same location of PARP1-mediated chromatin changes (Figs. 3, 4 and Additional file 1: Figs. S5 and S6), thus supporting our hypothesis that PARP1-mediated chromatin structural rearrangement regulates RNAPII elongation and splicing decisions.

## Discussion

Cotranscriptional removal of introns occurs in the vicinity of other gene expression machineries, including RNAPII and the chromatin remodeling factors. We previously documented that PARP1 is involved in splicing and proposed that PARP1 might play roles both in the ‘adaptor model’ and in the ‘kinetic model.’ Interestingly, these models are not mutually exclusive. We showed that PARP1 influences splicing, in part, through physical interactions of PARP1-bound chromatin, the spliceosome, and RNA. This finding supports the ‘adaptor model’ of cotranscriptional splicing [5, 11]. However, the effect of PARP1 on RNAPII elongation in the context of the ‘kinetic model’ remains unclear. At the beginning of this study, we proposed that PARP1 might also regulate RNAPII elongation. This concept is not unreasonable considering that several studies have shown that PARP1 influences RNAPII pausing at promoters [41], and PARP1 has an impact on negative elongation factor (NELF [42]) during RNAPII elongation. We therefore hypothesized that PARP1-bound chromatin regulates the RNAPII elongation rate by maintaining a specific chromatin structure, thus impacting splicing decisions. In this study, we undertook a comprehensive investigation of the influence of PARP1 on RNA polymerase elongation and splicing. Although most of this study focused on two target genes, our data indicate that PARP1-bound chromatin does influence splicing decisions. The local influence of chromatin was illustrated by the experiments on the two genes of study—*AKAP* and *CAPER*. These experiments showed that depending on the exon under scrutiny, a given chromatin factor has a variable effect favoring either exon inclusion or exclusion in a rather unpredictable manner.

Several mechanisms have been proposed to understand how the rate of RNAPII modulates alternative splicing. First, this process can occur through the phosphorylation state of the transcribing RNAPII [43] as well as the association of RNAPII with specific transcription factors [44]. Second, modulation can occur related to the effect of chromatin structure on the rate of elongation of the transcribing RNAPII, through DNA methylation [45] and histone modifications [12, 40, 46], which could create or eliminate chromatin roadblocks to elongation. The chromatin structure created impacts splicing decisions. For instance, increased nucleosome occupancy observed in exons compared to introns might aid in exon definition (reviewed in [47]) by modulating the RNAPII elongation rate. It is also possible that a chromatin context mediated by PARP1 represents a stumbling block that influences RNAPII elongation, thereby impacting the outcome of splicing.

The way that PARP1 affects the RNAPII elongation rate in vivo is not fully understood. It is possible that PARP1 acts together with other chromatin factors to remodel the chromatin structure. This could result in opening up the chromatin structure to influence RNAPII movement, which suggests that a certain degree of nucleosome remodeling is a prerequisite for, or a consequence of, active transcription. To investigate the role of PARP1 in transcriptional elongation and splicing in vivo, we measured the co-occupancy of PARP1 and RNAPII elongating polymerase (Fig. 2). PARP1-bound nucleosomes and RNAPII occupied similar regions in both genome-wide studies and in gene-specific loci.

We next determined the structure of the chromatin bound by PARP1 (Figs. 3, 4 and Additional file 1: Figs. S5 and S6). The nucleosomes bound by PARP1 had both activating and repressive marks within these two genes. Typically, the combination of repressive and activating histone marks at promoters, keeps genes expressed at low levels but poised for rapid activation. Additionally, RNAPII pause sites in transcription elongation often correlate with positioned nucleosomes [28, 35], and these nucleosomes are marked by bivalent histone marks. Thus, the fact that PARP1-bound nucleosomes within gene bodies displayed both activating and repressive marks indicated that this is a transcriptionally poised region. Most likely, PARP1-bound chromatin has created a paused region for transcriptional elongation. We posit these results are instigated directly by PARP1, as two siRNAs targeting different regions of PARP1 showed similar results, while PARylation inhibition had no effect producing results comparable to WT cells (Figs. 3, 4 and Additional file 1: Figs. S5 and S6). In fact, once PARP1 was depleted, the remodeling of chromatin structure to a more open chromatin structure via differential nucleosome occupancy/positioning, and histone modifications, would allow for transcriptional elongation (Fig. 3). Thus, our results showing bivalency at PARP1-occupied sites suggest these are sites of paused transcription, poised for activation. Another epigenetic mark related to alternative splicing regulation is DNA methylation. Though we analyzed possible differential DNA methylation changes at these sites (data not shown), the results were difficult to interpret. We attribute this difficulty to the lack and//or very low levels of DNA methylation present in the fly genome [48]. Overall, our data are consistent with the idea that a chromatin structure mediated by PARP1, impacts RNAPII elongation and possibly splicing decisions.

The effect of PARP1 on polymerase elongation and pausing has been shown in several previous studies. For instance, at heat shock promoters, immediately after induction, PARP1 mediates a rapid loss of nucleosomes in the bodies of induced Hsp70 genes, indicating that PARP1 acted as a block to transcription elongation. Immediately after the block is released through PARylation of PARP1 and histones, nucleosomes are remodeled, allowing for polymerase to move along the gene body with consequences in increased transcript levels [27, 41, 49]. Interestingly, another study showed that PARP1 PARylates and inhibits negative elongation factor (NELF), thus illustrating that PARP1 is important in transcription elongation. The latter study further showed that knockdown of PARP1 or inhibition of its PARylation activity, increased RNAPII pausing and reduced productive elongation [42]. These studies therefore provide functional links between PARP-1, ADP-ribosylation, and NELF. Furthermore, the binding of PARP1 to nascent RNA was shown to reduce the rate of RNA elongation by RNAPII, and subsequent automodification of PARP1 removes the transcriptionally inhibitory PARP1 molecules, thus up-regulating RNA synthesis [50]. Together, these studies suggest a link between PARP1 and RNAPII elongation and likely suggest that PARP1 initially would act as a “bump” to elongating RNAPII. With subsequent signals, PARP1 gets PARylated, releasing the block to transcription. PARP1 therefore could act as a hinge between signaling pathways and gene expression by communicating with the direct regulators of pausing [41, 42, 49]. Our data are consistent with the above studies. First, we observed that Pro-seq signals of paused RNAPII are in the same vicinity as PARP1-bound nucleosomes genome-wide (Additional file 1: Fig. S3). Sometimes the RNAPII signal is shifted a little downstream (Additional file 1: Fig. S3B–D), and the peak becomes broader with some tailing when compared to the PARP binding peak. These results hinted to the possibility that PARP1-bound nucleosomes could be involved in RNAPII elongation kinetics and possibly that some RNAPII backtracking occurs at these nucleosomes. RNAPII backtracking has been suggested to be the rate-limiting step in RNAPII elongation kinetics [35]. Although we could not definitively show RNAPII backtracking in this study, our results demonstrate a correlative presence of PARP1 nucleosome and RNAPII peaks within the gene body. Interestingly, knockdown of PARP1 resulted in loss of PARP1 signals at target regions with a corresponding loss of RNAPII signals (Fig. 2 and Additional file 1: Figs. S5 and S6), suggesting that loss of PARP-1 is necessary to achieve an efficient relieve from the elongation block of RNAPII into productive elongation.

However, these low-resolution studies do not provide a detailed understanding of kinetic coupling for the elongation rate, pausing, and nascent RNA structure. We therefore used a modified version of native elongating transcript sequencing (NET-seq), combining NET-seq [36] and 3′NT-seq [35] protocols. Although 3′NT maps all forms of RNAPII (paused, backtracked, and recovering), it does not map initiating RNAPII. Thus, all our subsequent analyses focused on the regions mapping to the body of the transcripts, as well as upstream and downstream of the TSS. Our analyses mapping the impact of PARP1 on the RNAPII location showed that some of the transcripts were lengthened after PARP1 knockdown while others were shortened. In comparing our NET-Seq data with our previous study on alternative splicing detected with PARP1-KD RNA-Seq [5] using rMATS [51], transcripts with shortened sites have twice as many alternative splice events than those with lengthened sites or with sites with no change, providing further evidence that pausing affects alternative splice mechanisms (see Additional file 2).

Based on our data, we propose a model whereby PARP1 mediates a chromatin structure (nucleosome remodeling and differential occupancies of histone marks) that slows down the rate of RNAPII elongation, resulting in enough resident time for RNAPII (Ser2P) and its associated splicing factors to recognize weak splice sites. And in the absence of PARP1, a more open chromatin structure ensues, allowing fast RNAPII elongation resulting in exon skipping (Fig. 6c). This model resonates with our results showing that after PARP1 knockdown, there is a loss of PARP1-RNAPII co-occupancy (fast RNAPII elongation) with consequences in exon skipping. In some cases though, we observed shortening of transcripts after PARP1 knockdown, indicative of a slowing down of polymerase elongation. Our model though cannot explain these instances of slow RNAPII and exon skipping in the absence of PARP1. Thus, further studies will be needed to tease how PARP1-bound chromatin, by slowing down the rate of RNAPII elongation, also results in exon exclusion. Generally, the ‘kinetic model’ of RNAPII’s effect on splicing decisions states that ‘slow RNAPII’ yields exons that are included while ‘fast RNAPII’ leads to exon skipping. However, contrary to this simplistic interpretation of the kinetic model, our results show that PARP1–chromatin binding instigates both lengthening and shortening of transcripts. We therefore interpret this effect of PARP1 on RNAPII elongation and splicing to be context specific. This interpretation is supported by our recent studies where we observed both exon inclusion and exon skipping after PARP1 depletion/PARylation inhibition [5, 11]. In addition, several studies that focused on the kinetic coupling of splicing and transcription showed that similar elongation changes promote different splicing outcomes [43, 52, 53]. Thus, changing the elongation rate may not alter the window of opportunity for positive splicing factors to bind but may also allow negative splicing factors to bind, justifying why slow RNAPII elongation can also favor exon skipping.

## Conclusions

We observed that PARP1 depletion produces skipping of exons on the *AKAP* and *CAPER* genes. RNAPII accumulated at the alternative exons of these genes, which are potential pause sites that facilitate changes in splicing [28, 54, 55]. Upon PARP1 depletion, we showed changes in alternative splicing events and RNAPII accumulation, suggesting that a correct chromatin structure is required for the normal splicing events taking place at these alternative exons. The salient question is, why is this region so sensitive to PARP1 depletion? We analyzed the context of PARP1 occupying this region (Figs. 3, 4; Additional file 1: Figs. S5 and S6). In the presence of PARP1, both activating and repressive histone marks are also present in this region. In addition to RNAPII elongation rate, PARP1-bound chromatin may also play a role in recruiting splicing factors. Because PARP1 can post-translationally modify histones [56, 57], it is possible that the absence of a correct nucleosomal structure under conditions of PARP1 depletion might also impair the recruitment of these splicing factors to the chromatin. Whatever the case, our data show a link in PARP1 depletion, RNAPII phosphorylation state, RNAPII elongation state, histone modification, and nucleosome positioning. In summary, the results in this study are consistent with the idea that PARP1 is crucial in gene regulatory processes in the cell.

## Materials

1. S2 cell culture and siRNA-mediated knockdown

*Drosophila melanogaster* S2 cells (obtained from Thermo Fisher Scientific, Waltham, MA 02451) were cultured in Schneider’s *Drosophila* medium (Life Technologies, Austin, TX 78744) supplemented with 10% heat-activated fetal bovine serum (Sigma, St Louis, MO 63146), 100 U/ml penicillin, and 100 μg/ml streptomycin at 25 °C. All experimental samples and controls were growth time and cell-density matched. siRNA-mediated PARP1 knockdown was performed as described previously [5]. siRNA1 for KD1 was made according to the Lis laboratory—Cornell University, Ithaca, NY, USA [49], while siRNA2 was made from PCR products targeting specific exons of PARP1 and LacZ were obtained from the Drosophila RNAi Screening Center (FlyRNAi.org—the database of the Drosophila RNAi screening center: 2012 update) to produce double-stranded RNA (dsRNA) for PARP1 knockdown and non-targeting control LacZ. Depletion of PARP1 was confirmed by Western blot and quantitative PCR using primers 1–4 (Additional file 1: Table S5).

2. PARylation inhibition

Cells were treated with 10 μM PJ34 hydrochloride (PARylation inhibitor, Thermo Fisher Scientific, #528150, Rockford, IL, 61101) or vehicle overnight for 16 h. Cells were then washed twice with PJ34-free medium, pelleted, and frozen for experiments.

3. Western blots

Western blots were performed using a standard protocol, and input dilutions were used as a quantitative indication of signal linearity. Protein samples were re-suspended in SDS loading buffer and electrophoresed on a 10% Tris–glycine gel with Tris running buffer. The proteins were transferred to PVDF membrane (Thermo Scientific, Rockford IL, 61101) and sequentially probed with primary antibodies for PARP1 and actin. Western blot-based detection was performed using alkaline phosphatase-coupled secondary antibodies (Sigma, St Louis, MO 63146) with Amersham ECF substrate for visualization (GE Healthcare, Waukesha, WI 53188), and images were obtained using Typhoon FLA 9500 (GE Healthcare, Piscataway, NJ 08854). ImageQuant TL software was used to quantify protein signals.

4. Measurement of poly-ADP-ribose (PAR) level

PAR assay in cellular extract was done using high-throughput chemiluminescent ELISA (HT PARP in vivo Pharmacodynamic Assay II kit from Trevigen, #4520-096-K, Gaithersburg, MD 20877). Net mean relative light units (RLU) values of the PAR standards were calculating by subtraction of the background (without PAR) from RLU values and presented as a function of PAR values (pg/ml).

5. Chromatin immunoprecipitation (ChIP)

Cross-link chromatin immunoprecipitation (X-ChIP) protocol was performed with slight modifications. In brief, 1 × 10^7^ cells were re-suspended in PBS and fixed with 1% formaldehyde for 10 min. Next, cells were washed 3 times with cold PBS and pelleted at 1200 rpm. The cell pellet was re-suspended in lysis buffer (50 mM HEPES–KOH (pH 7.5); 140 mM NaCl; 1 mM EDTA (pH 8); 1% SDS; 1% Triton X-100; 0.1% sodium deoxycholate and protease inhibitors). After 10 min incubation on ice, the lysate was sonicated for 20 min (30 s on/30 s off) with Bioruptor 300, (Diagenode, Sparta, NJ 07871) to shear DNA to an average fragment size of 150–700 bp. Cell debris was pelleted and the supernatant (containing chromatin) was used for immunoprecipitation (IP)—25 μg of chromatin was used in an IP experiment. Lysates containing chromatin were diluted 1:10 in RIPA buffer (50 mM mM Tris–HCl, (pH 8); 150 mM NaCl; 2 mM EDTA (pH 8); 1% NP-40; 0.1% SDS; 0.5% sodium deoxycholate and protease inhibitors), and 50 μl of chromatin was removed to serve as input. Primary antibodies (PARP1, S2P, 4H8, 8WG16, H3K4me3, and H3K27me3) were added to the samples (10 μg per 25 μg DNA) and rotated at 4 °C for 1 h. Rabbit IgG was used for negative or non-specific background control. The pre-bound antibody–chromatin complexes were incubated with Protein A/G Dynabeads (Thermo Fisher Scientific, Waltham, MA 02451) overnight at 4 °C with rotation in the presence of BSA (0.2 mg/ml). Using a magnetic stand for separation, all beads were washed twice with low salt buffer (0.1% SDS; 1% Triton X-100; 2 mM EDTA; 20 mM Tris–HCl (pH 8); 150 mM NaCl), then twice with high salt buffer (0.1% SDS; 1% Triton X-100; 2 mM EDTA; 20 mM Tris–HCl (pH 8); 500 mM NaCl). In addition, samples were washed twice with LiCl buffer [0.25 M LiCl; 1% NP-40; 1% sodium deoxycholate; 1 mM EDTA; 10 mM Tris–HCl (pH 8)]. Finally, specific DNA–protein complexes were eluted with 120 μl of elution buffer (1% SDS; 10 mM NaHCO_3_) for 15 min at 30 °C. The immunoprecipitated material and chromatin input were subjected to reverse cross-links according to Abcam X-ChIP protocol, and DNA was purified using the QIAquick PCR Purification Kit (Qiagen, Gaithersburg, MD 20878). Quantitative real-time PCR with primers 5–6, 9–10, 13–16, 43–44 (intron 4–5), 77–78 (intron 3–4) (Additional file 1: Table S5) was used to identify the level of specific DNA fragments from the immunoprecipitated DNA. All sets of primers were designed using Integrated DNA Technologies Primer Tools. Real-time, quantitative PCR (RT-qPCR) analysis was performed using CFX96 Real-Time System (Bio-Rad) with Taq DNA polymerase (MB042-EUT-10000, Syd Labs, Natick, MA 01760) and EvaGreen dye (Biotium). Reactions were performed at 25 μl and cycling parameters are as follows: 4 min at 94 °C, followed by 40 cycles of 45 s at 94 °C, 30 s at 60 °C and 60 s at 72 °C. For quality control purposes, melting curves for all samples were acquired (10 s at 95 °C and 60 s at 60 °C). For qPCR analysis, fold enrichment was measured against the IgG negative control and values were normalized to ChIP input.

6. Antibodies

For Western blot analysis:

Primary antibodies: PARP1 C terminal, rabbit (#39561, Active Motif, Carlsbad, CA 92008); Actin, mouse monoclonal (MA1-744, Thermo Fisher Scientific, Waltham, MA 02451). Secondary antibodies: anti-rabbit and anti-mouse IgG (whole molecule); alkaline phosphatase antibody (Sigma).

For ChIP:

PARP1, rabbit (#39561, Active Motif, Carlsbad, CA 92008); H5 (S2P), mouse monoclonal (ab24758, Abcam, Cambridge, MA 02139); 4H8 mouse monoclonal (ab5408, Abcam, Cambridge, MA 02139); 8WG16, mouse monoclonal (ab817, Abcam, Cambridge, MA 02139); H3K4me3, mouse monoclonal (ab1012, Abcam, Cambridge, MA, USA 02139); H3K27me3, mouse monoclonal (ab6147, Abcam, Cambridge, MA 02139), and for non-specificity control: Rabbit IgG (I8140; Sigma-Aldrich, St Louis, MO 63146).

7. PCR to measure isoform expression

Total RNAs were isolated from each sample using the RNeasy Plus Mini kit from Qiagen (Gaithersburg, MD 20878). 1 μg of RNA per reaction was used for reverse transcription reaction using iScript reverse transcriptase (Bio-Rad Laboratories, Hercules, CA 94547). The resultant cDNAs were used in PCR (S1000 Thermal Cycler, Bio-Rad) with the indicated primer sets (Additional file 1: Table S5: primers 5, 8, 9 and 12). PCR cycling parameters were as described in the *Chromatin Immunoprecipitation* section. PCR products were analyzed on 3.3% NuSieve agarose gel (Lonza, Rockland, ME 04841) with GelStar Nucleic Acid Gel Stain (Lonza, Rockland, ME, 04841) and visualized with Typhoon FLA 9500 (GE Healthcare, Piscataway, NJ 08854). ImageQuant TL software was used to quantify cDNA signals and calculate relative isoform expression. Splice isoforms were confirmed by cloning the products from PCR analyses using PCR Cloning Kit (NEB) according to the manufacturer’s protocol and sequenced by Eurofins Scientific.

8. Micrococcal (MNase) digestion of chromatin and DNA purification

Chromatin was digested at 27 °C using a predetermined concentration of MNase (Sigma-Aldrich). Digestion was stopped by adding 1/10th the volume of stop solution [10% of SDS and 0.5 mM EDTA (pH 8)]. Samples were further digested with RNAse A (Goldbio) and proteinase K (Sigma-Aldrich) to remove contaminating RNAs and proteins. DNA was finally purified as described in the *Chromatin Immunoprecipitation* section.

9. Nucleosome scanning analysis

The resulting purified DNA samples from MNase digestion were electrophoretically separated on 3.5% NuSieve agarose gel (Lonza), and mononucleosome-sized (140–200 bp) fragments were excised from the gel and purified using QIAquick PCR purification kit (Qiagen). Obtained DNA was analyzed using a ‘nucleosome walking’ technique. A set of overlapping primer pairs, each of which generate 100–120 bp PCR products that are located 20–40 bp away from neighboring primer pairs (Additional file 1: Table S5, primers 17–84), was used to analyze nucleosome positions. For every primer pair, the real-time PCR results (both of DNA isolated from nucleosomes and of naked DNA digested by micrococcal nuclease) were placed on a quantitative scale by comparison to serial dilutions of a known concentration of undigested genomic DNA, used as an absolute standard.

10. Genes of interest

*AKAP200* (Flybase ID: FBgn0027932, symbol: CG13388)

*CAPER* (Flybase ID: FBgn0031883, symbol: CG11266)

11. Net-seq 3′NT library preparation

Nascent RNA isolation was performed as described by Weber et al. [35], and cap selection was done as described by Ya-Lin Chiu et al. [58] using GFP-elF4E recombinant protein generously provided by Dr. G. Zentner, Indiana University. RNA:GFP-elF4E complexes were isolated using GFP-nAb Magnetic Agarose beads (Allele Biotech) according to the manufacturer’s protocol. Sequencing library preparation for nascent RNA samples was performed according to the Illumina Protocol with slight modifications. After 3′-SR adaptor ligation, RNA was fragmented to 30–100 nucleotides with the RNA Fragmentation Reagent (Albion, AM8740) and purified according to Mayer et al. [36]. In order to prevent RNA fragments ligation, we performed 3′-OH phosphorylation with T4 PNK (3′ phosphatase minus) before hybridization of reverse transcription primer and 5′-SR adaptor ligation. After reverse transcription with superscript reverse transcriptase (Invitrogen), we purified cDNA on 15% polyacrylamide TBE-urea gel to avoid primer dimer formation, excised the gel region between 50 and 300 nt and extracted cDNA. After PCR amplification, we performed quality control (QC) and size selection. To be confident, we cloned and sequenced the PCR products using Zero Blunt TOPO PCR Cloning kit (Thermo Fisher Scientific). Positive colonies were harvested; plasmid DNA was purified with QIAprep Spin Miniprep kit (Qiagen) and digested with EcoR1. Digested products were visualized on 1% agarose gel and sent for sequencing (Eurofins Scientific). After positive confirmation, accumulated cDNA was sent for sequencing by HiSeq 4000 Illumina platform.

12. Processing and alignment of sequencing reads

Nucleosome sequencing and analyses were done as in Matveeva et al. 2016 [5]. Details on processing and analyses of NET-seq are found in supplementary materials and methods.

## **Additional files**

**Additional file 1.** Supporting materials and methods section, including supplementary Figures 1–10 and supplementary Tables 1–6

**Additional file 2.** Western blot images of the full gel images found in Additional file 1: Supplementary Fig. S1

## Abbreviations

4H8: antibody to Ser5
8WG16: antibody to hypo-phosphorylated RNAPII
CTD: carboxy terminal domain
Dm6: *Drosophila* genome
GO:BP: gene ontology: biological process
GSE42117: PRO-seq data of transcriptionally engaged RNA polymerase
GSE56120: genome-wide data of PARP1 nucleosome occupancy
H3K4me3/H3K27me3: tri-methylated histone 3 at lysine 3/27
IP: immunoprecipitation
KD: knockdown
LW/HW: low/high molecular weight mRNA fragments
MNase: micrococcal nuclease
NELF: negative elongation factor
NET-seq: native elongating transcript sequencing
nt: nucleotide
PAR: poly-ADP-ribose
PARP1: poly [ADP-ribose] polymerase 1
PCR/RT-PCR: polymerase chain reaction/reverse transcription polymerase chain reaction
RNAPII: RNA polymerase II
RPKM: reads per kilobase of transcript = per million sequenced reads
SF3B1: splicing factor 3B1
TSS: transcription start sites
Upstream/downstream: gene location relative to the start codon
WT: wild type
X-ChIP: cross-link chromatin immunoprecipitation
