# Supplementary material for: Coupling of PARP1-mediated chromatin structural changes to transcriptional RNA polymerase II elongation and cotranscriptional splicing
Source: Epigenetics Chromatin. 2019 Feb 18;12:15. doi: 10.1186/s13072-019-0261-1 (PMC6378753; doi:10.1186/s13072-019-0261-1)
Supplement: Supplementary file 1 — Additional file 1. Supporting materials and methods section, including supplementary Figures 1–10 and supplementary Tables 1–6 [file 13072_2019_261_MOESM1_ESM.docx]

**1. Supplementary Materials and Methods**

Quality control **(**QC) of the raw sequence data was performed using FastQC **(**version 0.10.1) (1). The FastQC results indicated that the data was “quality value”, meaning that trimming for sequence quality was not necessary. The original fastq sequences were trimmed using Trimmomatic v0.33.to remove potential sequencing primers and adapters (2). The trimmed sequences were then collapsed to remove identical sequences which may be the result of PCR duplications using fastx_collapser v0.0.14 from the FASTX toolkit (3). Sequences for rRNA genes were downloaded from SILVA 132 (4) and tRNA genes were downloaded from GtRNAdb (5). The rRNA genes were further filtered to include only *Drosophila melanogaster* sequences using a custom script. A star genome index was generated from the combined rRNA and tRNA sequences using STAR v20201 (6). The trimmed sequences for each of the eight samples were then mapped against the rRNA and tRNA databases using star. Between 2-6% of the reads corresponded to tRNA or rRNA genes, which were then filtered out for subsequent analysis. Sample sequences were then aligned to the *Drosophila melanogaster* dm6 genome by aligning RNA filtered and collapsed reads mapping to a single location in the genome. After alignment, the resulting BAM files were split according to their strandedness using v1.3.1 (7). The forward and reverse BAM files were then converted to BED files using bedtools v2.26.0 (8) for further visualization in the UCSC genome browser (9). The BED files were then converted to WIG files using a custom script, only accounting for the very first base in the read, which allows for a single-base resolution of the stopping point of the ribosome. The WIG files were then converted to BIGWIG format for visualization in the UCSC genome browser using wigToBigWig v4. A custom UCSC track hub, consisting of a set of eight tracks **(**one for each sample) was created: <http://genome.ucsc.edu/cgi-bin/hgHubConnect?hgHub_do_redirect=on&hgHubConnect.remakeTrackHub=on&hgHub_do_firstDb=on&position=chr2L:8,415,690-8,431,297&hubUrl=http://kbrin.hsb.louisville.edu/~rouchka/star-filtered-uniquemapped-COLLAPSED/hub.txt>. The FlyBase gtf file Drosophila_melanogaster.BDGP6.91.chr.gtf was downloaded from the Ensembl ftp site and was separated into plus and negative strand genes. These strand-specific genes were then parsed using a custom script to produce regions for the five features **(**upstream, downstream, first 100 bp, last 100 bp, and transcript). A total of 100 bins were then created for each of the regions. Individual samples were then compared against the bins to determine if intersects occur for the bedtools and the counts for each individual bin within each individual feature for each of the samples using custom scripts. The counts for replicates were combined to further process differences between comparison groups.

**2. Comparison of NET-Seq with Alternative Splicing Data**

In comparing the alternative splicing detected with our PARP1-KD RNA-Seq data (10) using rMATS (11) to our NET-Seq data presented here, transcripts with shortened sites have twice as many (34 out of 307) alternative splice events than those with lengthened sites (23 out of 348) or with sites with no change (70 out of 1131), providing further evidence that pausing affects alternative splice mechanisms. For both the shortened (8 out of 307) and lengthened (7 out of 348) sites, retained intron events occur at a much higher rate than those with no change (10 out of 1131) (**Supplemental Table 6**). Interestingly, PARP itself seems to be regulated, with a shortened site corresponding to a skipped exon, thereby producing an alternative isoform. Genes with affected splice sites are listed below.

**SHORTENED**

**A3SS**: CWO, AXN, RECQ5, SRP

**A5SS**: PAR-1, VLC, BEST1, SU(F)

**MXE**: MFS17 (4 sites), TEP2 (4 sites), DHC64C

**RI**: ECT4, CG1646, RECQ5, CG9705, NTPASE (2 sites), CG1513, CG5080

**SE**: RHEB, SNX6, PARP, MFS17, CWO, MFS17, TSP97E, BAZ, ECT4

**LENGTHENED**

**A3SS**: CG3689

**A5SS**: RPS9

**MXE**: CAM, NAAM (2 sites)

**RI**: SAF-B (2 sites), CG3376, PMP70, CG7206, RPS14A, BRAT

**SE**: NFAT, CG40178, PPN (2 sites), MRP (6 sites), RL

**NO CHANGE**

**A3SS**: RPL3, MUD (2 sites), GUS, ENA, NOPP140, PITSLRE, CYCG, CNI, CIC

**A5SS**: AKAP200, TRL (3 sites), ZNT63C (2 sites), CG9331 (2 sites), RPL30, CHC

**MXE**: BSG (3 sites), RPS5A, CORA (2 sites), JUPITER, PS, CG17018, SPZ (2 sites), SCAF6, ZASP52

**RI**: CG3662, AKAP200, CG2201, KIS, CG7971, TRAF-LIKE, VSG, SSH, CG11695, CG42668

**SE**: TROL, AKAP200, PS (3 sites), MBC, CORA, GEL, EEF1DELTA, SQD, FAF, CTRIP, RTGEF, SIP2, GUS (3 sites), CG2765, NOPP140, BMM, SGG, PEP, RCD1, CG42674, GLO, STAI, MBS

**REFERENCES**

1. Andrews S. FastQC: A Quality Control Tool for High Throughput Sequence Data 2014 [Available from: <http://bioinformatics.babraham.ac.uk/projects/fastqc/>.

2. Bolger AM, Lohse M, Usadel B. Trimmomatic: a flexible trimmer for Illumina sequence data. Bioinformatics. 2014;30(15):2114-20.

3. Hannon G. FASTX-Toolkit 2014 [Available from: <http://hannonlab.cshl.edu/fastx_toolkit/>.

4. Quast C, Pruesse E, Yilmaz P, Gerken J, Schweer T, Yarza P, et al. The SILVA ribosomal RNA gene database project: improved data processing and web-based tools. Nucleic acids research. 2013;41(Database issue):D590-6.

5. Chan PP, Lowe TM. GtRNAdb 2.0: an expanded database of transfer RNA genes identified in complete and draft genomes. Nucleic acids research. 2016;44(D1):D184-9.

6. Dobin A, Davis CA, Schlesinger F, Drenkow J, Zaleski C, Jha S, et al. STAR: ultrafast universal RNA-seq aligner. Bioinformatics. 2013;29(1):15-21.

7. Li H, Handsaker B, Wysoker A, Fennell T, Ruan J, Homer N, et al. The Sequence Alignment/Map format and SAMtools. Bioinformatics. 2009;25(16):2078-9.

8. Quinlan AR, Hall IM. BEDTools: a flexible suite of utilities for comparing genomic features. Bioinformatics. 2010;26(6):841-2.

9. Karolchik D, Barber GP, Casper J, Clawson H, Cline MS, Diekhans M, et al. The UCSC Genome Browser database: 2014 update. Nucleic acids research. 2014;42(Database issue):D764-70.

10. Matveeva E, Maiorano J, Zhang Q, Eteleeb AM, Convertini P, Chen J, et al. Involvement of PARP1 in the regulation of alternative splicing. Cell Discov. 2016;2:15046.

11. Park JW, Jung S, Rouchka EC, Tseng YT, Xing Y. rMAPS: RNA map analysis and plotting server for alternative exon regulation. Nucleic acids research. 2016;44(W1):W333-8.

**Supplementary Figures**

**** **Supplementary Figure 1**. **Measurement of PARP1 and PARylation levels in Drosophila S2 cells**. (**A**) Relative PARP1 mRNA level change as measured by RT-PCR **(**top) and (B) western blot analysis of PARP1 protein **(**bottom). Consistent knockdown results were obtained using two siRNAs targeting different regions of *PARP1* gene (siRNA1 and siRNA 2) as we had previously described ^5^. Knockdown experiments and relative mRNA level changes as measured by RT-PCR were performed in triplicate. (C) PAR levels in WT, KD and PJ34 treated cells were measured using a pharmoacodynamics Assay kit (Trevigen). Results are presented as mean ± SD with p-value < 0.05 **(**Student’s *t*-test method).

**Supplementary Figure 2**: **Validation of PARP1’s effect on splicing at *AKAP200* and *CAPER* genes using a second siRNA.** PCR products with exon junction spanning primers were ran on 4% nusieve agarose gel images show the difference in splicing products between non-treated (WT), PARP1 knockdown (KD) using siRNA 1 (KD1) and siRNA2 (KD2) cells. Additionally PARylation inhibition (PJ34) had no effect on the splicing pattern at these two genes are also shown. Actin is shown as a PARP1 non-target gene.

**Supplementary Figure 3**. **RNAPII stalls near PARP1-nucleosome gene regions.** Shown are metagene plots for PARP1-nucleosome binding, total nucleosome positioning and RNAPII location as measured by Pro-seq. At the exons found at the beginning of genes **(A**), the RNAPII signal overlaps that of PARP1-nucleosome binding. **(B**) At the ends of first exons, although signals for both PARP1 and RNAPII are lower, the PARP1 is shifted slightly to the left of RNAPII binding signal. **(C**) Strong signal of PARP1-nuclosome binding and RNAPII at the beginning of skipped exons and **(D**) lower signals at the ends of skipped exons.

**Supplementary Figure 4. Effect of PARP1 on RNAPII elongation**. The occupancy of **(A**) PARP1 and different forms of RNAPII ChIP-qPCR on PARP1 target genes *AKAP200* and *CAPER.* **(B**) Ser2P occupancy for elongating RNAPII. **(C**) 8WG16 occupancy for preinitiation sites RNAPII. **(D**) 4H8 occupancy for initiating and first regions of the gene RNAPII. Blue bars represent none-treated cells **(**WT) and red bars – PARP1 knockdown **(**KD). All experiments were performed in triplicates and results are presented as mean ± SD. P-values **(**Student’s *t*-test method) are shown below the charts.

**Supplementary Figure 5. PARP1 KD using siRNA2 confirmed the effect of PARP1 on RNAPII occupancy, while PARylation had not effect.** The occupancy of **(A**) PARP1 and different forms of RNAPII ChIP-qPCR on PARP1 target genes *AKAP200* and *CAPER.* **(B**) Ser2P occupancy for elongating RNAPII. **(C**) 8WG16 occupancy for pre-initiation sites RNAPII. **(D**) 4H8 occupancy for initiating and first regions of the gene RNAPII. Blue bars represent none-treated cells **(**WT), red bars – PARP1 knockdown **(**KD), green bars – PARylation inhibition (PJ34-treated cells). All experiments were performed in triplicates and results are presented as mean ± SD. P-values **(**Student’s *t*-test method) are shown below the charts.

**Supplementary Figure 6. Nucleosome repositioning in PARP1 KD cells and not in PARylation inhibited cells.** Nucleosome repositioning in Figure 3 using PARP1 siRNA1 is confirmed using PARP1 siRNA2. Depletion of PARP1 using siRNA2, confirmed the results of nucleosome repositioning in Figure 3, using PARP1 siRNA1. Inhibition of PARP1’s enzymatic activity using PJ34, showed no difference in nucleosome positioning relative to WT conditions. Results are mean ± SD of 3 independent experiments and differences were considered significant as measured by Student’s *t*-test (p < 0.05).

**Supplementary Figure 7. PARP1-mediated dynamic occupancy of histone marks H3K4me3 and H3K27me3.** KD of PARP1 results in differential histone PTM occupancy while PARylation inhibition had no effect. KD of PARP1 using siRNA2 confirmed the differential histone PTM occupancy in Figure 4. ChIP-qPCR experiments showed that KD resulted in increased occupancy of H3K4me3 at both AKAP (A) and CAPER (B) with a concordant decrease in H3K27me3 occupancy at bot AKAP (C) and CAPER (D). In contrast, compared to WT cells, PARylation inhibition had no impact. Experiments were performed in triplicates and are represented as mean ± SD. *Experimental differences were considered significant as measured by Student’s *t*-test when p < 0.05.

**Supplementary Figure 8.** **Difference in transcriptional elongation between wild type and PARP knockdown conditions**. Metagene analyses of NET-seq maps showing examples of RNAPII locations at different gene regulatory regions. Line graphs provide examples of NET-seq reads in upstream and transcript regions **(A** and **B**), downstream regions **(C)** and at the early **(**first 100 bp), and late genes bodies **(**last 100 bp) **(D** and **E**). The red arrows and the red bar show regions with significant changes.

**Supplementary Figure 9**. **Examples of shortened (A) and lengthened (B) genes due to PARP1 knockdown.** Distribution of reads from PARP1-KD vs. WT cells with significantly differentially expressed gene regions **(**p < 0.01).

**Supplementary Figure 10**. **Metagene analysis of NET-seq at different genomic regions for wild type (WT) and PARP1 knockdown (KD) cells.** The data reveal shortened transcripts in the upstream **(A**), downstream **(C**), the first 100 bp **(E**) and the last 100 bp **(G**) regions **(**orange lines - WT, red lines - KD). Lengthened transcripts were identified in the upstream **(B**), downstream **(D**),–the first 100 bp **(F**) and the last 100 bp regions **(H**) **(**purple and violet lines – WT, blue lines – KD).

**Supplementary Tables**

**Supplementary Table 1. Top 20 genes with shortened transcripts.**

| REGION | GENE | GENE_SYMBOL | P-VALUE | Difference |
| --- | --- | --- | --- | --- |
| chrX:19852214-19859379 | FBgn0031078 | Nup205 | 0.047619 | 1 |
| chr3R:9006456-9008030 | FBgn0037653 | CG11982 | 1.70E-05 | 0.909091 |
| chr3L:15509445-15510837 | FBgn0028377 | gdl-ORF39 | 0.004761 | 0.833333 |
| chrX:20248309-20251093 | FBgn0031098 | CG17068 | 0.000714 | 0.777778 |
| chr2L:19584235-19586440 | FBgn0032814 | CG10366 | 0.003932 | 0.75 |
| chr3L:13483533-13486280 | FBgn0036372 | Abp1 | 0.020979 | 0.75 |
| chr3L:15520318-15521554 | FBgn0262689 | CR43159 | 1.30E-05 | 0.734848 |
| chrX:12018640-12020719 | FBgn0030364 | Lsm12 | 0.002258 | 0.684211 |
| chr2L:2311680-2358944 | FBgn0031414 | eys | 0.034965 | 0.675 |
| chr3R:21219077-21222843 | FBgn0038870 | Oga | 0.003932 | 0.666667 |
| chr2L:138384-140992 | FBgn0051976 | ovm | 0.014985 | 0.666667 |
| chr3R:3322810-3354486 | FBgn0086917 | spok | 0.035714 | 0.666667 |
| chr2R:5804712-5860831 | FBgn0085414 | dpr12 | 0.042986 | 0.666667 |
| chr2R:7493806-7496466 | FBgn0033183 | CG1620 | 0.000709 | 0.622222 |
| chrX:12717624-12730953 | FBgn0264678 | CR43963 | 0.014705 | 0.615385 |
| chr3R:10093761-10101356 | FBgn0263413 | CR43459 | 0.000104 | 0.609524 |
| chrX:4648592-4650032 | FBgn0025615 | Torsin | 0.001905 | 0.609091 |
| chr2R:9587193-9587989 | FBgn0285949 | RpL31 | 1.00E-05 | 0.6 |
| chr2L:3462218-3466112 | FBgn0005616 | msl-2 | 0.043956 | 0.6 |
| chr2R:18167255-18168380 | FBgn0260401 | MED9 | 5.00E-06 | 0.597884 |

**Supplementary Table 2. Top 20 genes with lengthened transcripts.**

| REGION | GENE | GENE_SYMBOL | P-VALUE | Difference |
| --- | --- | --- | --- | --- |
| chr3L:5780051-5784685 | FBgn0044419 | Pmi | 0.003076 | -1 |
| chr2R:12877094-12880230 | FBgn0013305 | Nmda1 | 0.035714 | -1 |
| chrX:8404535-8407263 | FBgn0030035 | PIG-T | 0.035714 | -1 |
| chr3L:8372405-8391171 | FBgn0001253 | ImpE1 | 0.00923 | -0.95833 |
| chr2R:23972431-23975408 | FBgn0002791 | mr | 0.008791 | -0.91667 |
| chrX:644761-646386 | FBgn0025634 | CG13367 | 2.00E-05 | -0.9 |
| chrX:21374002-21385159 | FBgn0003423 | slgA | 0.001998 | -0.9 |
| chrX:22980722-23004648 | FBgn0016975 | stnB | 6.10E-05 | -0.875 |
| chr3R:25340252-25344838 | FBgn0027376 | rha | 0.047619 | -0.8 |
| chr3R:29749219-29754528 | FBgn0260990 | yata | 0 | -0.79352 |
| chr4:436957-443911 | FBgn0039907 | lgs | 0.006993 | -0.75 |
| chr3R:21105046-21107972 | FBgn0266674 | Sec15 | 0.000173 | -0.71795 |
| chr2R:9116122-9122000 | FBgn0003612 | Su(var)2-10 | 0.002704 | -0.71429 |
| chrX:10086527-10148876 | FBgn0083940 | RhoU | 1.50E-05 | -0.71282 |
| chrX:13718734-13721977 | FBgn0030514 | CG9941 | 5.90E-05 | -0.70588 |
| chr2L:8529141-8541439 | FBgn0028394 | CG17834 | 0.007647 | -0.67857 |
| chr3L:19298623-19337609 | FBgn0003089 | pip | 8.00E-06 | -0.65942 |
| chr2R:20242752-20245388 | FBgn0034488 | CG11208 | 6.60E-05 | -0.65686 |
| chr3R:28820221-28824508 | FBgn0039602 | CG1647 | 0.00017 | -0.65 |
| chr2R:8444560-8447164 | FBgn0014469 | Cyp4e2 | 0.003264 | -0.63333 |

**Supplementary Table 3. Top 20 enriched GO:BP for genes with shortened transcripts.**

| GO_ID | GO_DESCRIPTION | P-VALUE | FDR |
| --- | --- | --- | --- |
| GO:0006898 | receptor-mediated endocytosis | 2.91E-06 | 0 |
| GO:0050803 | regulation of synapse structure or activity | 3.66E-06 | 0 |
| GO:0040007 | growth | 4.96E-06 | 0 |
| GO:0006897 | endocytosis | 5.97E-06 | 0 |
| GO:0048589 | developmental growth | 6.76E-06 | 0 |
| GO:2000756 | regulation of peptidyl-lysine acetylation | 2.42E-05 | 0 |
| GO:1901983 | regulation of protein acetylation | 2.42E-05 | 0 |
| GO:0035065 | regulation of histone acetylation | 2.42E-05 | 0 |
| GO:0044723 | single-organism carbohydrate metabolism | 3.74E-05 | 0 |
| GO:0040008 | regulation of growth | 3.85E-05 | 2.02E-04 |
| GO:0010256 | endomembrane system organization | 4.01E-05 | 1.96E-04 |
| GO:0031056 | regulation of histone modification | 5.39E-05 | 3.77E-04 |
| GO:0007416 | synapse assembly | 5.45E-05 | 3.74E-04 |
| GO:0031667 | response to nutrient levels | 6.22E-05 | 3.64E-04 |
| GO:0051246 | regulation of protein metabolic process | 6.42E-05 | 3.60E-04 |
| GO:0009991 | response to extracellular stimulus | 6.91E-05 | 3.57E-04 |
| GO:0061024 | membrane organization | 8.64E-05 | 5.26E-04 |
| GO:0050808 | synapse organization | 9.05E-05 | 5.22E-04 |
| GO:0030258 | lipid modification | 9.96E-05 | 0.001367521 |
| GO:0009891 | positive regulation of biosynthetic process | 1.13E-04 | 0.001322314 |

**Supplementary Table 4. Top 20 enriched GO:BP for genes with lengthened transcripts.**

| GO_ID | GO_DESCRIPTION | P-VALUE | FDR |
| --- | --- | --- | --- |
| GO:0051960 | regulation of nervous system development | 5.13E-06 | 0 |
| GO:0045595 | regulation of cell differentiation | 3.90E-06 | 0 |
| GO:0000226 | microtubule cytoskeleton organization | 1.87E-07 | 0 |
| GO:0006935 | chemotaxis | 4.88E-06 | 0 |
| GO:0048667 | cell morphogenesis involved in neuron differentiation | 1.22E-06 | 0 |
| GO:0009968 | negative regulation of signal transduction | 2.88E-07 | 0 |
| GO:0048585 | negative regulation of response to stimulus | 3.95E-07 | 0 |
| GO:0023057 | negative regulation of signaling | 9.95E-07 | 0 |
| GO:0010648 | negative regulation of cell communication | 2.83E-07 | 0 |
| GO:0007409 | axonogenesis | 1.99E-06 | 0 |
| GO:0061564 | axon development | 3.55E-06 | 0 |
| GO:0030178 | negative regulation of Wnt signaling pathway | 2.38E-06 | 0 |
| GO:0007051 | spindle organization | 8.65E-06 | 2.41E-04 |
| GO:0097485 | neuron projection guidance | 1.06E-05 | 4.55E-04 |
| GO:0006897 | endocytosis | 9.56E-06 | 4.65E-04 |
| GO:2000026 | regulation of multicellular organismal development | 3.41E-05 | 5.61E-04 |
| GO:0051298 | centrosome duplication | 3.39E-05 | 5.66E-04 |
| GO:0040007 | growth | 3.34E-05 | 5.71E-04 |
| GO:0007411 | axon guidance | 2.66E-05 | 6.00E-04 |
| GO:0007052 | mitotic spindle organization | 2.44E-05 | 6.12E-04 |

**Supplementary Table 5**. **Primers used in the study.**

| Gene | # | Primer name | Direction | Primer sequence (5'-3') |
| --- | --- | --- | --- | --- |
| *PARP1* | 1 | PARP1/F | Forward | TCGACGTGTCGTGGATGTGAACAA |
| *PARP1* | 2 | PARP1/R | Reverse | ACAAAGGTTGGCCTCCGTACTTCA |
| *Actin* | 3 | Actin5C/F | Forward | TCGCGATTTGACCGACTACCTGAT |
| *Actin* | 4 | Actin5C/R | Reverse | TTGATGTCACGGACGATTTCACGC |
| *AKAP* | 5 | AkEx4/F | Forward | CTGCTGCTGGTGAGGATATAA |
| *AKAP* | 6 | AkEx4/R | Reverse | GTCCTTCTTGCCAAAGGAAATG |
| *AKAP* | 7 | AkEx6/F | Forward | GATCTCGCCAAGGATCTGAA |
| *AKAP* | 8 | AkEx6/R | Reverse | GAGTAGGATTATTCGCATGTAACG |
| *CAPER* | 9 | CapEx3/F | Forward | CCCGAATTGCAGCGAAGTA |
| *CAPER* | 10 | CapEx3/R | Reverse | TTATCCGGCCTTTGGGAAC |
| *CAPER* | 11 | CapEx8/F | Forward | GTGGACACGATGACGACTAC |
| *CAPER* | 12 | CapEx8/R | Reverse | GGGATCGTTTGCTTTGCTT |
| *AKAP* | 13 | AkEx5/F | Forward | AGTTGAAGCCAAGTCCGTAG |
| *AKAP* | 14 | AkEx5/R | Reverse | TCCACAATAACGGACTCGAAC |
| *CAPER* | 15 | CapEx4/F | Forward | TGTCTTTGCAGAGAATCGAATAAG |
| *CAPER* | 16 | CapEx4/R | Reverse | CAGCTTCTGGCACATTCAAA |
| *AKAP* | 17 | A1/F | Forward | GCGGTAGAGGTAAGCAAATCA |
| *AKAP* | 18 | A1/R | Reverse | CTCAGAATGCTCCTCAGTTTCC |
| *AKAP* | 19 | A2/F | Forward | AGGAAACTGAGGAGCATTCTG |
| *AKAP* | 20 | A2/R | Reverse | TTCAGCGACCGCATCAC |
| *AKAP* | 21 | A3/F | Forward | TGATGCGGTCGCTGAAA |
| *AKAP* | 22 | A3/R | Reverse | CCTTCTTGGACTTGGACTTGA |
| *AKAP* | 23 | A4/F | Forward | CTGCTGCTGGTGAGGATATAA |
| *AKAP* | 24 | A4/R | Reverse | CCTTCTTGGACTTGGACTTGA |
| *AKAP* | 25 | A5/F | Forward | GCAAGAAGGACAAACAGAAACC |
| *AKAP* | 26 | A5/R | Reverse | GCCATTTGTAGCCACCGA |
| *AKAP* | 27 | A6/F | Forward | ACAAATGGCGAGGCTGAA |
| *AKAP* | 28 | A6/R | Reverse | GTGGCAGTGGCAGTCTTAT |
| *AKAP* | 29 | A7/F | Forward | CTAAATGTTATAAGACTGCCACTGC |
| *AKAP* | 30 | A7/R | Reverse | GGCCTGTTTCTCCTGCTC |
| *AKAP* | 31 | A8/F | Forward | GCCAACGGAGAAACCGAAA |
| *AKAP* | 32 | A8/R | Reverse | GTTCTTCGGCGGGCTTG |
| *AKAP* | 33 | A9/F | Forward | AAGAACCAGCCACAGTCAC |
| *AKAP* | 34 | A9/R | Reverse | TGTCCATTGGTCACCACTTC |
| *AKAP* | 35 | A10/F | Forward | GGTGACCAATGGACATGGA |
| *AKAP* | 36 | A10/R | Reverse | CATCAACGTTGCTCGGAATG |
| *AKAP* | 37 | A11/F | Forward | TTCCGAGCAACGTTGATGAC |
| *AKAP* | 38 | A11/R | Reverse | CCTCAATTTGCTGTCCCTTCT |
| *AKAP* | 39 | A12/F | Forward | GGGACAGCAAATTGAGGTAAAC |
| *AKAP* | 40 | A12/R | Reverse | GTGCAGTGTTCTCTGTGATTTC |
| *AKAP* | 41 | A13/F | Forward | CAACCGAAATCACAGAGAACAC |
| *AKAP* | 42 | A13/R | Reverse | TTGGGTCTGAACTAACTCTGC |
| *AKAP* | 43 | A14/F | Forward | AGAGTTAGTTCAGACCCAAACA |
| *AKAP* | 44 | A14/R | Reverse | GGGTCACTGTTTCAATCACTTC |
| *AKAP* | 45 | A15/F | Forward | CCAGCAGTGAAGTGATTGAAAC |
| *AKAP* | 46 | A15/R | Reverse | CTGCTAACCGCCTTGATGA |
| *AKAP* | 47 | A16/F | Forward | GCAATCATCAAGGCGGTTAG |
| *AKAP* | 48 | A16/R | Reverse | TGGACACAGGAGCAACAA |
| *AKAP* | 49 | A17/F | Forward | TTGTTGCTCCTGTGTCCA |
| *AKAP* | 50 | A17/R | Reverse | TGGCTTCAACTTCGGGAAT |
| *AKAP* | 51 | A18/F | Forward | AGTTGAAGCCAAGTCCGTAG |
| *AKAP* | 52 | A18/R | Reverse | TCCACAATAACGGACTCGAAC |
| *CAPER* | 53 | C1/F | Forward | TTTCTCCAAACGTGCCAGA |
| *CAPER* | 54 | C1/R | Reverse | TCAAAGTCCTCGGCCATATTC |
| *CAPER* | 55 | C2/F | Forward | CCCGAATTGCAGCGAAGTA |
| *CAPER* | 56 | C2/R | Reverse | TTATCCGGCCTTTGGGAAC |
| *CAPER* | 57 | C3/F | Forward | GAGGCGCCGTACATGAAA |
| *CAPER* | 58 | C3/R | Reverse | CCTTGGGTGGGCACAAA |
| *CAPER* | 59 | C4/F | Forward | GCCCACCCAAGGCAATTA |
| *CAPER* | 60 | C4/R | Reverse | CGAAGACGACTTACATACAAACAC |
| *CAPER* | 61 | C5/F | Forward | ACAGGCGTACGTGCATTT |
| *CAPER* | 62 | C5/R | Reverse | AAGACCACTTAGTATGCATCGAC |
| *CAPER* | 63 | C6/F | Forward | GTATGTAAGTCGTCTTCGTCTCG |
| *CAPER* | 64 | C6/R | Reverse | ACGCAGATTGGCGTTCC |
| *CAPER* | 65 | C7/F | Forward | CGCCAATCTGCGTGCTG |
| *CAPER* | 66 | C7/R | Reverse | TTTCCGTTGGCGAAACACT |
| *CAPER* | 67 | C8/F | Forward | CACACTAGACTGCGTTCATACA |
| *CAPER* | 68 | C8/R | Reverse | GTATCAAATGCCGCGTAGGA |
| *CAPER* | 69 | C9/F | Forward | TACTGCAGTGGGTGTACAGT |
| *CAPER* | 70 | C9/R | Reverse | CATGACTGTCTTGTTAGTAATTCAATCC |
| *CAPER* | 71 | C10/F | Forward | TTGTAGCAATTGGTATTCCTCT |
| *CAPER* | 72 | C10/R | Reverse | CAAGTATGATTTCATATGGCTGAG |
| *CAPER* | 73 | C11/F | Forward | TTTCGGTGTGTTGTGGAATAAA |
| *CAPER* | 74 | C11/R | Reverse | CCAACTTTAGCTGGATGTCATAG |
| *CAPER* | 75 | C12/F | Forward | AGTAGTCTATGACATCCAGCTAAAG |
| *CAPER* | 76 | C12/R | Reverse | GTGAGACAGCAGATAGGAAGAAA |
| *CAPER* | 77 | C13/F | Forward | TCATGAGTTCAACGCTCCTATTT |
| *CAPER* | 78 | C13/R | Reverse | GGTGATTGTGTGTGGGTAGTT |
| *CAPER* | 79 | C14/F | Forward | ACCCACACACAATCACCATTAA |
| *CAPER* | 80 | C14/R | Reverse | CTCTAGCAACAATATTGAGCTTATTCG |
| *CAPER* | 81 | C15/F | Forward | TGTCTTTGCAGAGAATCGAATAAG |
| *CAPER* | 82 | C15/R | Reverse | CAGCTTCTGGCACATTCAAA |
| *CAPER* | 83 | C16/F | Forward | CACAATCCAATTGAAATCGTTACCT |
| *CAPER* | 84 | C16/R | Reverse | CCCAGACAAGTTAGGAGGAGTA |

**Supplementary Table 6.**  **Alternative splice events detected for genes with NET-Seq data.** A3SS: alternative 3’ splice site; A5SS: alternative 5’ splice site; MXE: mutually exclusive exon; RI: retained intron; SE: skipped exon.

|  | A3SS | A5SS | MXE | RI | SE | TOTAL |
| --- | --- | --- | --- | --- | --- | --- |
| Lengthened (n=348) | 1 | 1 | 3 | 7 | 11 | 23 |
| Shortened (n=307) | 4 | 4 | 9 | 8 | 9 | 34 |
| No change (n=1131) | 10 | 10 | 13 | 10 | 27 | 70 |
