# Supplementary material for: Coupling of PARP1-mediated chromatin structural changes to transcriptional RNA polymerase II elongation and cotranscriptional splicing
Source: Epigenetics Chromatin. 2019 Feb 18;12:15. doi: 10.1186/s13072-019-0261-1 (PMC6378753; doi:10.1186/s13072-019-0261-1)

Coupling of PARP1-mediated chromatin structural changes to transcriptional RNA polymerase II elongation and cotranscriptional splicing

Elena A. Matveeva[1], Qamar M. H. Al-Tinawi[1,2], Eric C. Rouchka[3,4] , Yvonne N. Fondufe-Mittendorf[1]*

[1]Department of Molecular and Cellular Biochemistry, University of Kentucky, Lexington, KY 40536, USA; [2]Alfaisal University, Al Maather' Riyadh, 12714 Saudi Arabia; [3]Kentucky Biomedical Research Infrastructure Network Bioinformatics Core, 522 East Gray Street, Louisville, KY 40292, USA; [4]Department of Computer Engineering and Computer Science, University of Louisville, Louisville, KY 40292, USA

EAM: elmatva@uky.edu

QMHA-T: qaltinawi@alfaisal.edu

ECR: eric.rouchka@louisville.edu

YNF-M: y.fondufe-mittendorf@uky.edu

*Corresponding author: Yvonne N. Fondufe-Mittendorf

Western blot images of cutout gels in Additional file 1: Supplementary Figure S1 and full agarose gel image of cutout gels in Aditional File 1: Supplemental Figure S2

Full gel images of PARP1 and actin supporting Supplemental Figure 1

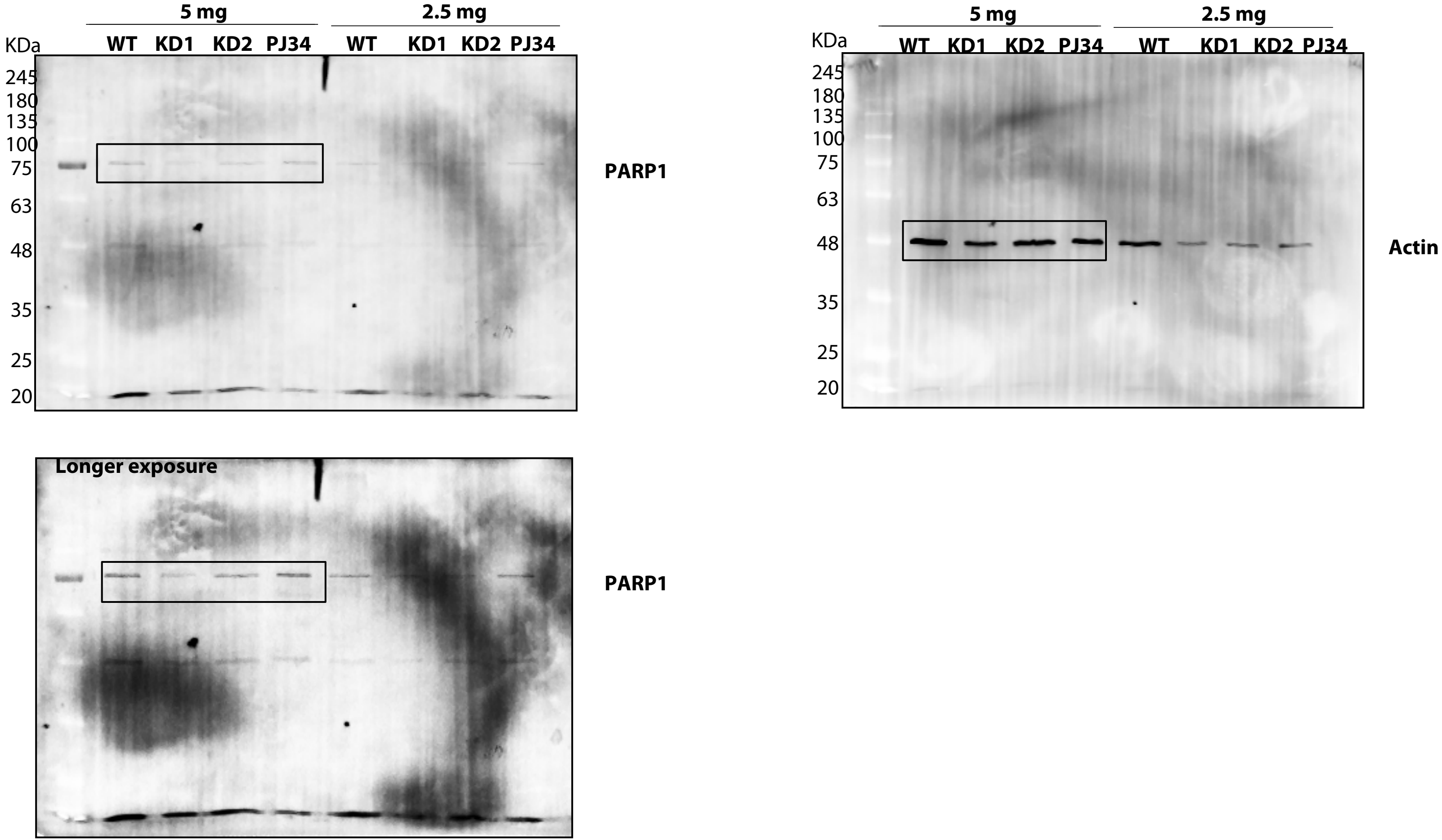

Full agarose gel image of splicing isoforms supporting Supplemental Figure 2

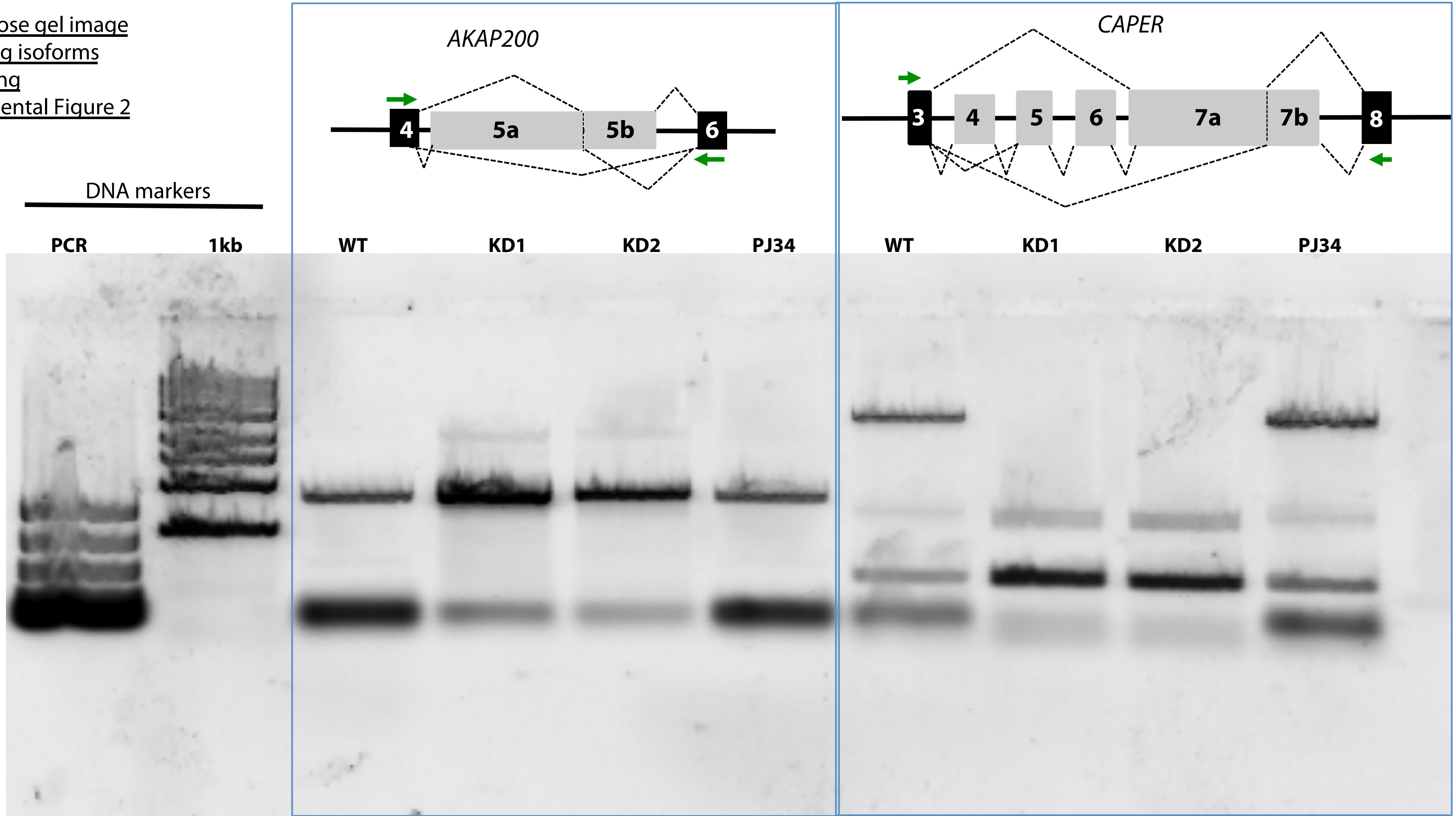

Supplement: Supplementary file 2 — Additional file 2. Western blot images of the full gel images found in Additional file 1: Supplementary Fig. S1 [file 13072_2019_261_MOESM2_ESM.pdf]
